# *In vitro* competition with *Bifidobacterium* strains impairs potentially pathogenic growth of *Clostridium perfringens* on 2′-fucosyllactose

**DOI:** 10.1080/19490976.2025.2478306

**Published:** 2025-03-18

**Authors:** Aruto Nakajima, Aleksandr A. Arzamasov, Mikiyasu Sakanaka, Ryuta Murakami, Tomoya Kozakai, Keisuke Yoshida, Toshihiko Katoh, Miriam N. Ojima, Junko Hirose, Saeko Nagao, Jin-Zhong Xiao, Toshitaka Odamaki, Dmitry A. Rodionov, Takane Katayama

**Affiliations:** a Graduate School of Biostudies, Kyoto University, Kyoto, Japan; b Infectious and Inflammatory Disease Center, Sanford Burnham Prebys Medical Discovery Institute, La Jolla, CA, USA; c Innovative Research Institute, Morinaga Milk Industry Co, Ltd, Zama, Kanagawa, Japan; d Department of Food and Nutrition, Kyoto Women’s University, Kyoto, Japan; e Nagao Midwife Clinic, Kyoto, Japan

**Keywords:** *Clostridium perfringens*, Bifidobacteria, 2'-fucosyllactose, α-toxin

## Abstract

Fortifying infant formula with human milk oligosaccharides, such as 2'-fucosyllactose (2'-FL), is a global trend. Previous studies have shown the inability of pathogenic gut microbes to utilize 2'-FL. However, the present study demonstrates that the type strain (JCM 1290^T^) of *Clostridium perfringens*, a pathobiont species often more prevalent and abundant in the feces of C-section-delivered infants, exhibits potentially pathogenic growth on 2'-FL. The expression of genes for α-toxin, an activator of NLRP3 inflammasome, and ethanolamine ammonia-lyase, a factor responsible for the progression of gas gangrene, was significantly upregulated during 2'-FL assimilation compared to growth on lactose. However, colony-forming unit of *C. perfringens* JCM 1290^T^ markedly decreased when co-cultivated with selected strains of *Bifidobacterium*, a taxon frequently detected in the breastfed infant gut. Moreover, during co-cultivation, the expression of virulence-related genes, including the gene for perfringolysin O – another activator of NLRP3 inflammasome – were significantly downregulated, while the lactate oxidation genes were upregulated. This can occur through two different mechanisms: direct competition for 2'-FL between the two organisms, or cross-feeding of lactose, released from 2'-FL by *C. perfringens* JCM 1290^T^, to *Bifidobacterium*. Attenuation of α-toxin production by the selected *Bifidobacterium* strains was observed to varying extents in 2'-FL-utilizing *C. perfringens* strains clinically isolated from healthy infants. Our results warrant detailed *in vivo* studies using animal models with dysbiotic microbiota dominated by various types of *C. perfringens* strains to further validate the safety of 2'-FL for clinical interventions, particularly on vulnerable preterm infants.

## Introduction

The gut microbiota established during the early stages of life is considered to have long-lasting effects on host health^1^ and breastfeeding is instrumental to the development of a healthy microbiota in the infant gut. Among other various functional components, human milk contains oligosaccharides with a degree of polymerization of ≥3 (termed human milk oligosaccharides, HMOs) as the third largest solid component after lactose and lipids.^2,3^ Diverse functions that have been reported for HMOs include inhibition of pathogen binding to intestinal epithelial cells,^4^ alleviation of inflammation by inhibiting toll-like receptor 4 signaling,^5,6^ and stimulation of the growth of beneficial gut microbes like *Bifidobacterium* species.^7,8^ Infant gut-associated bifidobacteria are equipped with varied gene sets dedicated to the assimilation of HMOs, depending on species and strains,^9^ and through inter-species competition and/or inter-species facilitation, they frequently dominate the infant gut microbial community.^10,11^ With increasing awareness of the importance of the infant gut microbiota, fortifying infant formula with HMOs has attracted much attention. 2'-Fucosyllactose (2'-FL) is among the first commercially available HMOs. The safety of 2'-FL administration is supported by several clinical studies^12–14^ and previous reports indicating that pathogenic gut microbes do not assimilate this sugar.^15–17^ Indeed, the HMO consumption ability has only been reported for several *Bacteroides* species,^18,19^ phylotypes of *Akkermansia*,^20^ and several species belonging to a butyrate-producing *Roseburia-Eubacterium* group,^21^ apart from *Bifidobacterium* species.

*Clostridium perfringens*, an opportunistic pathogen, is more frequently and abundantly detected in the feces of premature neonates delivered by C-section than those of infants delivered vaginally at full-term.^22,23^ Although the causal relationship is controversial,^24^ the presence of *C. perfringens* in the gut, especially those carrying the perfringolysin O (PFO) gene (*pfoA*),^25^ is considered to be associated with the incidence or exacerbation of necrotizing enterocolitis (NEC), a severe disease with high mortality.^26^ Alpha-toxin (PLC) and PFO, major known toxins of *C. perfringens*, are shown to activate NLRP3 inflammasome in human cell lines and a mouse model.^27^ *C. perfringens* possesses a repertoire of glycoside hydrolases (GHs) active on host glycans^28–30^; however, earlier studies have failed to demonstrate the efficient utilization of HMOs by this species.^15–17^ In the present study, we show that *C. perfringens* strains, including clinical isolates from healthy infants, can assimilate 2'-FL to varying degrees, ranging from partial to complete consumption. Notably, the avid consumption of 2'-FL by *C. perfringens* JCM 1290^T^ was associated with the upregulation of virulence-related genes. We also show that this potentially harmful growth was impaired by co-cultivation with *Bifidobacterium* strains *in vitro*, which is accompanied by cell death and down-regulation of the virulence-related genes. The levels of α-toxin (PLC) in the supernatants were reduced, albeit to varying extents, when the type strain and certain clinical *C. perfringens* strains were co-cultivated with the selected *Bifidobacterium* strains. While our study focused on *in vitro* experiments and does not demonstrate *in vivo* pathogenic behavior of this pathobiont, the results indicate that caution may be warranted when administering 2'-FL to vulnerable populations.

## Materials and methods

### Chemicals

2'-FL was the gift from Glycom A/S (Hørsholm, Denmark). Porcine gastric mucin (PGM) was obtained from Sigma Aldrich (St. Louis, MO, USA). 2-Anthranilic acid (2-AA) and sodium cyanoborohydride were purchased from Nacalai Tesque (Kyoto, Japan) and Sigma Aldrich, respectively. Maltoheptaose was from Toronto Chemical (Ontario, Canada). A mixture of HMOs was purified from pooled breastmilk collected from Japanese mothers as described previously.^11^ All other chemicals used were of analytical grade.

### Bacterial strains and culture conditions

*C. perfringens* JCM 1290^T^, JCM 3816, JCM 3817, and JCM 3818, *Bifidobacterium longum* subsp. *longum* (*B. longum*) JCM 1217^T^, JCM 7052, JCM 7054, and JCM 7056, *Bifidobacterium breve* JCM 1192^T^, JCM 7016, JCM 7019, and JCM 7020, *Bifidobacterium bifidum* JCM 1209, JCM 1254, JCM 1255^T^, and JCM 7002, and *Bifidobacterium longum* subsp. *infantis* (*B. infantis*) JCM 1210, JCM 1222^T^, JCM 1272, and JCM 11344 were obtained from the Japan Collection of Microorganisms (RIKEN Bioresource Center, Tsukuba, Japan). *B. longum* MCC10007, *B. breve* MCC1851, *B. bifidum* MCC2030, and *B. infantis* MCC1872 were obtained from the Morinaga Culture Collection (Morinaga Milk Industries, Co. Ltd., Zama, Japan). Eight *C. perfringens* strains were isolated from feces collected from eight distinct healthy infants aged 1–5 months. Diluted fecal suspensions were spread on Gifu Anaerobic Medium (GAM, Nissui Pharmaceutical, Tokyo, Japan) or Tryptose Sulfite Cycloserine (TSC, Merck, Burlington, MA, USA) agar plates, and the plates were incubated at 37°C under anoxic conditions with an AnaeroPack (Mitsubishi Gas Chemicals, Tokyo, Japan). Colonies with gliding motility on GAM agar plates or colonies with black pigment on TSC agar plates were picked and subjected to the 16S rRNA gene sequencing using a primer pair of 8F (5'-agagtttgatcctggctcag-3') and 1492 R (5'-ggttaccttgttacgactt-3'). The amplicon sequences of the eight distinct clinical isolates, named SCM023, SCM041, SCM044, AN673, AN679, AN680, AN685, and AN689, shared >99.3% identity with that of JCM 1290^T^.

Yeast and Casitone medium (YC), which is a fatty acid-free version of YCFA medium,^31^ Reinforced Clostridial Medium (RCM), GAM, and de Man-Rogosa-Sharpe broth (MRS) were used for the cultivation of bacteria. Sugars in the original medium formulations were replaced with either fucose (Fuc), lactose (Lac), 2'-FL, a mixture of Fuc and Lac with a molar ratio of 1:1, or a mixture of Fuc, galactose (Gal), and glucose (Glc) with a molar ratio of 1:1:1. Bacterial growth in a 96-well plate was continuously monitored by measuring OD_605_ using Byonoy Absorbance 96 Plate Reader (Byonoy GmbH, Hamburg, Germany). When cultivation was conducted in test tubes, samples were taken at the indicated time points, and the OD_600_ was measured by a spectrophotometer (Multiskan GO, Thermo Fisher Scientific, Waltham, MA, USA). Thin-layer chromatography was used for qualitative analysis of residual sugars in culture supernatants.^32^ *Escherichia coli* DH5α, a host strain for genetic manipulations, was grown in LB broth (Becton Dickinson, Franklin Lakes, NJ, USA). Antibiotics were used at the following concentrations (μg/mL) when necessary: chloramphenicol (Cm), 5 for *E. coli* and 2.5 for bifidobacteria; ampicillin (Amp), 100 for *E. coli*.

### *Toxinotyping and genotyping of* C. perfringens *strains*

The toxinotypes of the four JCM strains and the eight clinical *C. perfringens* isolates were examined using the multiplex PCR method described by Rood *et al*.^33^ The *afc3* (1,2-α-l-fucosidase), *pfoA*, and *eutB* genotypes were examined by genomic PCRs using the following primer pairs: 5'-tgagtccaaaactgtgcagc-3' and 5'-gctatgggatgggaatttga-3' for *afc3*,^34^ 5'-atgtaatgtactttgaaacaggacaagg-3' and 5'-ttgatagttaagcattacgtcatctcca-3' for *eutB*,^35^ and 5'-caagtattgcaatggctttatgtctg-3' and 5'-ctttataagagctttgaaagcagcttg-3' for *pfoA*.^36^

### *Preparation of recombinant Afc3 (1,2-α-l-fucosidase) from* C. perfringens

The catalytic domain (28–866 amino acid residues) of Afc3 (CPF_RS10355) of *C. perfringens* JCM 1290^T^ was predicted by InterPro.^37^ The corresponding DNA region was amplified using a primer pair of 5'-AAGGAGATATACATATGtctagtagatttaatgaagtag-3' and 5'-GGTGGTGGTGCTCGAGttttacatcttcagctatta-3', and the fragment was inserted into the *Nde* I and *Xho* I sites of pET-23b (+) to generate a C-terminally hexahistidine-tagged protein. An In-Fusion HD Cloning kit (Takara Bio, Shiga, Japan) was used for ligation (capital letters in the primer sequences represent the region for In-Fusion recombination). After sequence confirmation, the resulting plasmid was introduced into *E. coli* BL21(DE3) Δ*lacZ* carrying pRARE2.^38^ The transformants were grown in LB medium containing Amp and Cm at 18°C to OD_600_ ≈0.4, and the recombinant protein expression was induced by adding 0.1 mM isopropyl-β-d-1-thiogalactopyranoside. Following further incubation for 21 h, the cells were harvested and suspended in a buffer consisting of 50 mM HEPES, 300 mM NaCl, and 10 mM imidazole (pH 8.0). After the disruption of the cells by ultrasonication, the soluble fraction was applied onto Ni-NTA Spin Columns (QIAGEN, Hilden, Germany). The recombinant protein was purified according to the manufacturer’s instructions. The eluates were then combined and concentrated using an Amicon Ultracel-30K centrifugal filter (Merck, Burlington, MA, USA), during which the buffer was exchanged with 20 mM Tris-HCl (pH 8.0). The protein was further purified by MonoQ 5/50 GL anion-exchange column chromatography (GE Healthcare, Chicago, IL, USA). The elution was performed by a linear gradient of 0–0.5 M NaCl in 20 mM Tris-HCl (pH 8.0). The purity of the recombinant protein was evaluated by sodium dodecyl sulfate-polyacrylamide gel electrophoresis. The purified fractions were combined and stored at 4°C after the addition of Tween-20 at the final concentration of 0.1 mg/mL. The protein concentration was determined spectrophotometrically at 280 nm using a theoretical absorption coefficient of 177,620 M^−1^ cm^−1^ calculated based on the amino acid sequence.

### Enzyme assay

1,2-α-l-Fucosidase activity was determined by measuring the amount of Fuc released from the substrates. l-Fuc was quantified by a high-performance anion-exchange chromatography system with pulsed amperometric detection (HPAEC-PAD) or by a fucose dehydrogenase-coupled reaction, as described previously.^28,39^ The standard reaction mixture contained 50 mM McIlvaine buffer (pH 6.0), 1 mM 2'-FL as a substrate, and 0.15 nM purified enzyme. The reaction was carried out at 37°C for 5 min, in which the linearity of the reaction rate was observed. The kinetic parameters were calculated by curve-fitting the experimental data to the Michaelis-Menten equation using KaleidaGraph (Synergy Software, Reading, PA, USA).

### Targeted gene disruption

The fucose dehydrogenase gene of *B. longum* MCC10007 (*fumC*) and the fucose permease gene of *B. breve* MCC1851 (*fucP*), which are the most upstream genes in the Fuc assimilation pathways of the respective strains,^11,40,41^ were inactivated by single-crossover recombination events (Supplementary Figure S1a, b). In brief, the internal regions of *fumC* and *fucP* were amplified by PCR using primer pairs of 5'-ACGTTTCATGAATTAcaagggcataacgctgcgac-3' and 5'-GCCTAAGCGCCATTAcggccacaacgacggcattg-3', and of 5'-ACGTTTCATGAATTAacgcgttgaactcgccgttc-3' and 5'-GCCTAAGCGCCATTAccacgaccagcacaacg-3', respectively. Each DNA fragment was then inserted into the *Nsi*I site of a suicide plasmid pMSK209^28^ by In-Fusion HD Cloning kit (Takara Bio). After sequence confirmation, the resulting plasmids were introduced into respective strains by electroporation.^7^ Following recovery culture for 3 h at 37°C, transformants were spread on GAM agar plates containing Cm. Integration of the plasmid into the *fumC* and *fucP* loci of respective strains was verified by genomic PCRs using primers that were designed to anneal outside of the region used for recombination (Supplementary Figure S1c). The primer pairs used were: 5'-acggagatgaagctcgac-3' and 5'-ttccgaagtctcggtcatg-3' for the *fumC* locus and 5'-ccacttctcgctaggcaatatc-3' and 5'-tgagcaggccgaagaaggag-3' for the *fucP* locus. Phenotypic changes of the obtained mutants were assessed based on their growth characteristics and sugar consumption ability (Supplementary Figure S1d, e).

### *Isolation of revertants of the* B. longum fumC *mutant*

Revertants of the *fumC* mutant of *B. longum* MCC10007 were obtained by incubating the mutant in MRS-CS supplemented with 0.5% (*w/v*) 2'-FL in the absence of the antibiotic pressure. After multiple passages to enrich the revertant population, dilutions of the culture were spread on an antibiotic-free GAM agar plate to allow colony formation. Three colonies that showed sensitivity to Cm were selected, and genomic rearrangements at the *fumC* locus as well as their growth properties were confirmed (Supplementary Figure S1f, g). One of the three isolates was used to assess its phenotypic recovery.

### *Co-cultivation of* C. perfringens *strains with* Bifidobacterium *strains*

*C. perfringens* JCM 1290^T^ was co-cultivated with *Bifidobacterium* strains in a one-to-one combination. YC medium supplemented with 0.5% (*w/v*) 2'-FL was used for cultivation. Overnight mono-cultures of *C. perfringens* JCM 1290^T^ and *Bifidobacterium* strains were separately harvested by centrifugation and washed twice with sugar-free YC medium, and the resulting suspensions were used for inoculation. The initial culture (at 0 h of incubation) contained 5.6–6.8 × 10^6^ CFU/mL of *C. perfringens* JCM 1290^T^ and 3.5–12 × 10^5^ CFU/mL of each *Bifidobacterium* strain. CFU of *C. perfringens* JCM 1290^T^ was determined by spreading serially diluted cultures on GAM agar plates and TSC agar plates both containing 400 μg/mL of d-cycloserine, to which all the bifidobacterial strains showed sensitivity. Colonies that appeared on GAM agar plates were considered to represent the vegetative cells, while those that appeared on TSC agar plates were considered to represent both vegetative and spore cells. We omitted the heat treatment process prior to plating the diluted cultures on TSC agar, as the spores of the type strain are reported to be heat-labile.^42^ The spore cell counts were estimated by subtracting the CFU obtained on GAM plates from that obtained on TSC plates. Bifidobacterial CFU was measured using MRS agar plates containing mupirocin (50 μg/mL) and norfloxacin (15 μg/mL), to which *C. perfringens* JCM 1290^T^ was sensitive. MRS was supplemented with sodium ascorbate (3.4 mg/mL) and l-cysteine-HCl (0.3 mg/mL) before use. Co-cultivations of three clinical *C. perfringens* strains (SCM023, SCM041, and SCM044) with *B. longum* MCC10007 or *B. breve* MCC1851 were performed with the initial CFU of the *C. perfringens* strains (5.4–7.4 × 10^6^ CFU/mL) being approximately 10-fold higher than that of the *Bifidobacterium* strain (3.7–6.3 × 10^5^ CFU/mL).

### Transcriptome analysis

Mono-cultivated and co-cultivated *C. perfringens* JCM 1290^T^ was subjected to transcriptome analysis with three biological replicates. Samples were taken when the OD_605_ values reached 0.7 (for mono-culture) or post 6- and 12 h inoculation (for co-culture) and centrifuged. The resulting pellets were immediately suspended in 400 μL of TE buffer [10 mM Tris-HCl and 1 mM EDTA (pH 8.0)], to which 800 μL of RNAprotect Bacterial Reagent (Qiagen) was added. After incubation for 5 min at room temperature, cells were harvested again and stored at − 80°C until use. RNA was isolated using a RNeasy Mini kit (Qiagen) according to the manufacturer’s instructions, except that the cells were incubated with 15 mg/mL of lysozyme (Sigma Aldrich), 1 kU/mL of mutanolysin (Sigma Aldrich), and 1.5 mg/mL of proteinase K (Qiagen) in 200 μL of TE buffer before disruption. Library preparation was performed using an Illumina Stranded Total RNA Prep, Ligation with Ribo-Zero Plus kit and IDT for Illumina RNA UD Indexes Set A-B Ligation (Illumina, San Diego, CA, USA) following the manufacturer’s protocol. Oligonucleotide pools for depleting the rRNA genes of *C. perfringens*, *B. longum*, and *B. breve* were designed by Illumina and synthesized by Integrated DNA Technologies (San Diego, CA). The quality and concentration of adapter-tagged sequence library were measured by the Agilent High Sensitivity DNA kit and the Agilent RNA 6000 Nano kit (Agilent Technologies, Santa Clara, CA, USA), respectively. Libraries were sequenced on the NextSeq 1000 system with the NextSeq 1000/2000 P2 Reagent kit (Illumina) using 50-bp paired-end reads. Quality trimming and removal of Illumina sequencing adapters were performed via fastp (v0.22.3).^43^ Trimmed reads, consisting of both bifidobacterial and clostridial cDNA transcripts, with a mean ± standard deviation (SD) of 16,097,877 ± 10,803,286 counts, were paired and pseudoaligned to the *C. perfringens* ATCC 13124^T^ (= JCM 1290^T^) transcriptome (GenBank accession No. NC_008261.1) using Kallisto (v0.48).^44^ Consequently, a mean ± SD of 6,037,745 ± 4,879,438 read pairs were mapped to *C. perfringens* transcripts. The two replicates collected post 12 h co-cultivation with *B. longum* MCC10007 yielded 858,657 and 991,528 read pairs, respectively, while for the other samples, more than 1 million read pairs were obtained (Supplementary Table 1). All following analyses were performed using R (v4.2.1)^45^ in RStudio (v2023.03.0 Build 386)^46^ and Bioconductor (v3.16).^47^ Kallisto output was read into the R environment using the *tximport* package^48^ and normalized by the trimmed mean of *M* values (TMM) method using the *edgeR* package.^49^ Genes with counts per million of >1 in ≥3 samples were extracted. To identify differentially expressed genes (DEGs), precision weights were first applied to each gene based on its mean-variance relationship using the *voom* function in the *limma* package.^50^ Then, DEGs were identified via linear modeling and Bayesian stats using *limma* (adjusted *p* value < 0.01, |log_2_ fold change| > 1). The false discovery rate was calculated by the Benjamini-Hochberg procedure.^51^ Volcano plots and principal component analysis (PCA) plots were produced using *ggplot2*.^52^ Genes were annotated based on a RAST^53^-annotated version of the *C. perfringens* ATCC 13124^T^ genome, which was additionally subjected to extensive manual curation in mcSEED, a private clone of the publicly available SEED platform for gene annotation.^54^ Glycoside hydrolases (GHs) presumed to be involved in mucin *O*-glycan degradation were extracted in reference to the previous report.^29^

### Semi-quantification of α-toxin and determination of sialidase activity in the culture supernatants

Alpha-toxin in the culture supernatants were semi-quantified using a Multiscreen AgELISA Alpha toxin and *Clostridium perfringens* kit (Bio-X Diagnostics, Rochefort, Belgium) according to the manufacturer’s instructions. A standard sigmoidal curve was created using GraphPad Prism (v10.2.1) using the serially diluted control material appended to the kit. Sialidase activity of culture supernatants was measured using 1% (*w/v*) PGM as a substrate. Sodium acetate (pH 5.5) was used for a buffer at 60 mM concentration. After incubation at 37°C for 1 min, twice volume of 1 M Na_2_CO_3_ was added to stop the reaction. Released *N*-acetylneuraminic acid (NeuAc) was quantified using HPAEC-PAD as described previously.^28^

### Instrumental analyses

#### Alcohol detection

For detecting 1-propanol, a high-performance liquid chromatography (HPLC) system (Gilson, Middleton, WI, USA) equipped with an Aminex HPX-87H column (7.8 mm × 300 mm, Bio-Rad Laboratories, Hercules, CA) was used. The elution was performed at a flow rate of 0.7 mL/min at 50°C with 5 mM H_2_SO_4_ and was monitored using a RID-20A refractive index detector (Shimadzu, Kyoto, Japan). The standard curve was created using known concentrations of 1-propanol. For the detection of 1,2-propanediol and 1,3-propanediol, the same HPLC system but equipped with an InertSustain AQ-C18 column (4.6 mm × 250 mm, GL Sciences, Tokyo, Japan) was used. The elution was performed as described previously.^55^ Culture supernatant of *C. perfringens* JCM 1290^T^ grown in YC medium supplemented with 1% (*w*/*v*) Lac or 2'-FL was injected.

#### Organic acid detection

Acetate, butyrate, formate, *iso*-valerate, lactate, propionate, pyruvate, succinate and valerate were quantified using a Dionex ICS-3000 system (Thermo Fisher Scientific) as described previously.^56^ Known concentrations of respective acids were used to create standard curves.

#### HMO detection

HMO consumption by *C. perfringens* strains were analyzed by the previously described method involving fluorescence labeling with 2-AA in the presence of sodium cyanoborohydride.^57^ YC medium supplemented with 1% (*w/v*) of an HMO mixture purified from pooled breastmilk was used for cultivation. Maltoheptaose was used as an internal standard for quantification.

### Statistical analysis

Two-tailed Student’s *t*-test or Tukey’s Honest Significant Difference test following one-way analysis of variance test (ANOVA) was carried out using GraphPad Prism (v10.3.0) to assess statistical significance. When necessary, the false discovery rate was calculated by the Benjamini-Hochberg procedure.^51^ *p* values of <0.05 were considered statistically significant.

## Results and discussion

**Figure 1.**
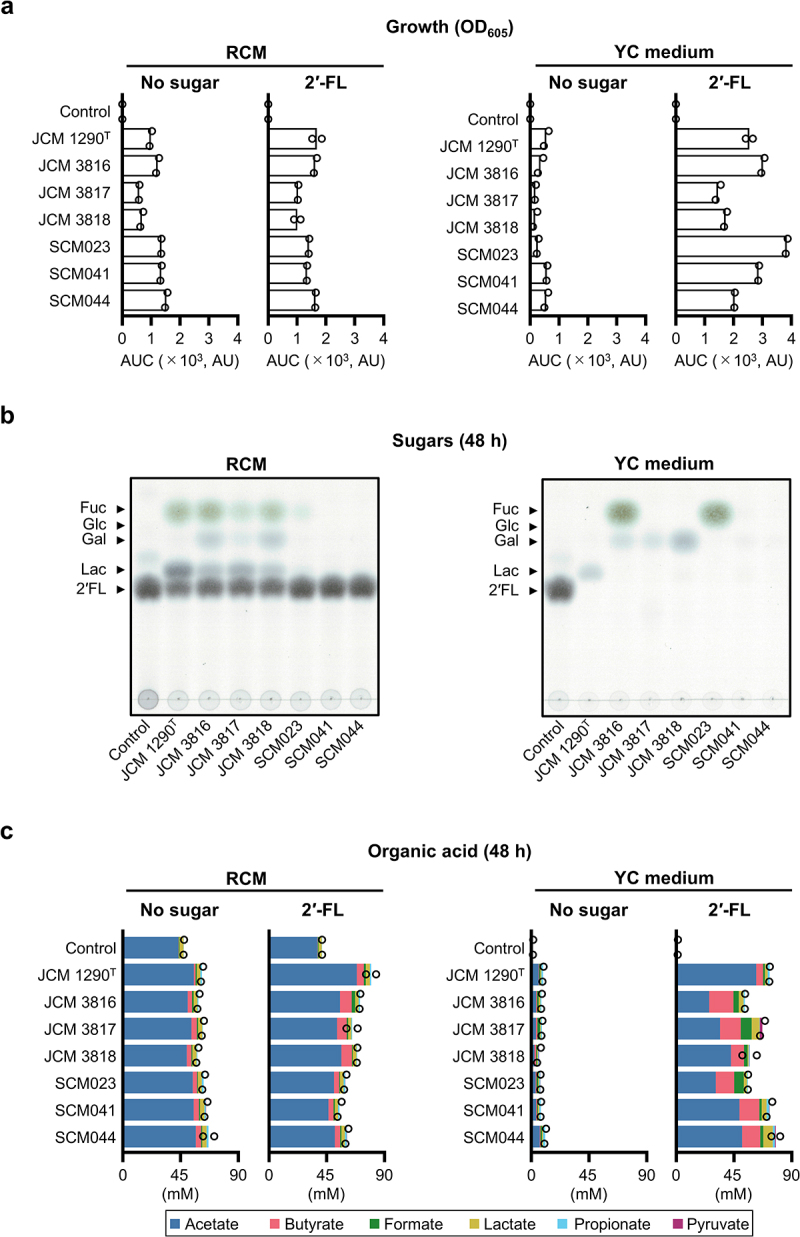
*C. perfringens* strains assimilate 2'-FL in YC medium. a, growth of four JCM and three clinical *C. perfringens* strains cultivated in RCM (left panel) and YC medium (right panel) supplemented without or with 1% 2'-FL. The growth is represented by the area under the curve (AUC, arbitrary unit) (see supplementary figure 3a). Bacteria-free medium was used as a control. Bars represent the mean of two independent experiments, with each data point shown by a circle. b, TLC analysis of sugars in supernatants of *C. perfringen*s strains grown on 2'-FL for 48 h. The results shown are representative of two independent experiments. c, organic acid production post 48 h cultivation. Concentrations of organic acids are represented by stacked bar charts with acetate, butyrate, formate, lactate, propionate, and pyruvate shown in blue, pink, green, khaki, cyan, and purple, respectively. Neither valerate, *iso*-valerate, nor succinate was detected. Bars represent the mean of two independent experiments, with each data point indicating the total amount of detected organic acids (see supplementary figure 3b for the concentrations of individual organic acids). Samples obtained in (a) were used for the analysis. See the source data file for the individual data points corresponding to the graphs (a and c).

Previous studies consistently showed limited utilization of 2'-FL by *C. perfringens*,^15–17^ even though this species appears to possess the genetic machinery to digest host glycans.^30^ We first hypothesized that its limited ability to utilize 2'-FL stems from the low activity of its extracellular 1,2-α-l-fucosidase (CPF_RS10355, *afc3*) toward H-antigens (Fucα1-2 Galβ-*O*-R),^34,58^ as the enzyme has a methionine replacement at the conserved glutamate residue that is crucial for the recognition of the Gal moiety of H-antigens.^59^ However, a purified recombinant 1,2-α-l-fucosidase (Afc3) from *C. perfringens* JCM 1290^T^ showed a high catalytic efficiency for 2'-FL (*k*_cat_/*K*_m_ = 8.9 × 10^2^ s^−1^ mM^−1^) (Supplementary Figure S2). Its *K*_m_ value (1.2 mM ≈ 0.06% [*w*/*v*]) is sufficiently low for hydrolyzing 2'-FL added to the medium and found in breastmilk (~3 mM).^57^ The limited 2'-FL assimilation ability of *C. perfringens* JCM 1290^T^ was reproduced when Reinforced Clostridial Medium (RCM), in which Glc was replaced with 2'-FL, was used for cultivation. Growth of the type strain and the other strains, specifically JCM 3816, JCM 3817, JCM 3818, and three clinical isolates (SCM023, SCM041, and SCM044), plateaued within 12 h cultivation regardless of the presence or absence of 2'-FL (Supplementary Figure S3a). However, when we used Yeast and Casitone medium (YC medium), which is frequently utilized for fecal cultivation, all strains consumed 2'-FL and produced more organic acids compared to when grown in RCM (Figure 1 and Supplementary Figure S3). Acetate and butyrate were the two main short-chain fatty acids produced by the fermentation of 2'-FL. Neither *iso-*valerate, succinate, nor valerate was detected. tBlastn analysis revealed a high prevalence of *afc3* in the complete genomes of *C. perfringens*, with 111 out of 123 strains harbouring the gene. However, 5 genomes of the 111 strains contain frameshift mutations at the locus. The four JCM strains and three clinical isolates (SCM023, SCM041, and SCM044) also degraded and assimilated other HMOs to different degrees when grown on YC medium supplemented with an HMO mixture purified from pooled milk (Supplementary Figure S4). The utilization of released fucose (Fuc) varied among the strains (Figure (1b) and Supplementary Figure S4b). We additionally isolated five *C. perfringens* strains from distinct infants and examined their ability to utilize 2'-FL. The five strains, all of which were genotypically *afc3*-positive (Supplementary Figure S5), utilized 2'-FL, although two (AN680 and AN689) out of the five strains exhibited a low ability to ferment the sugar (Supplementary Figure S6). The other three strains (AN673, AN679, and AN685) avidly consumed 2'-FL, with a varied Fuc assimilation phenotype. These results indicate that many *C. perfringens* strains have a notable potential to assimilate 2'-FL. The toxinotypes of the eight clinical isolates were determined to be type A by multiplex PCR analysis, and thus these isolates were genotypically all *plc*-positive (Supplementary Figure S5). Genotyping analysis revealed that, while all clinical isolates were positive for *eutB* (described later), two out of eight strains were *pfoA*-negative. Toxinotypes determined for the four JCM strains were consistent with those reported previously.^60^

**Figure 2.**
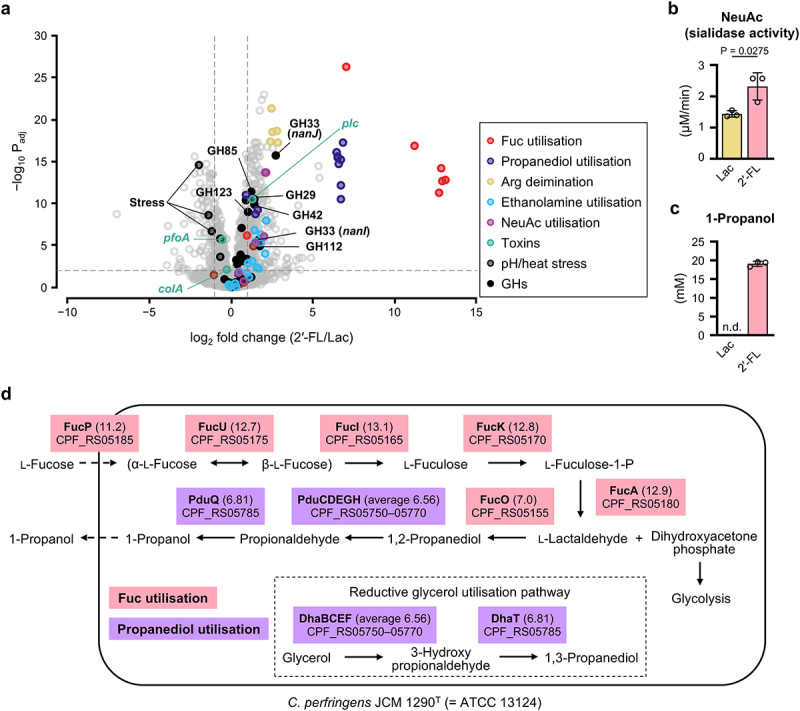
Elevated expression of virulence-related genes in *C. perfringens* JCM 1290^T^ grown on 2'-FL. a, a volcano plot comparing the normalized gene expression values of cells grown on 2'-FL versus those grown on Lac. Fold changes (log_2_) and adjusted *p* values (−log_10_) obtained by multiple *t*-tests with false discovery rate correction were used for plotting. *C. perfringens* JCM 1290^T^ was used for the analysis. Selected genes are shown in different colors with their functions or names. RNA-seq data from three biological replicates were used for the analysis (see supplementary tables 1 and 2). b and c, sialidase activity and the concentration of 1-propanol in supernatants of *C. perfringens* JCM 1290^T^ grown in YC medium supplemented with 1% Lac (≈29 mM) or 2'-FL (≈20 mM). Supernatants collected when the cells were harvested for (a) were used for measuring NeuAc-releasing activity (b). PGM was used as a substrate. 1-propanol was quantified using the supernatant post 24 h cultivation (c) (see supplementary figure 7). Bars and whiskers represent the mean ± standard deviation (SD) of three independent experiments, with each data point shown by a circle. Statistical significance was evaluated by two-tailed *t*-test (b). d, the metabolic pathway for Fuc utilization in *C. perfringens* JCM 1290^T^ reconstructed in the mcSEED database.^54^ Fuc is split into lactaldehyde and dihydroxyacetone phosphate after isomerization and phosphorylation in the cytoplasm. While the latter is sent to glycolysis, the former is converted to 1-propanol. Fuc transporter and catabolic enzymes are shown with their locus tags and common names. Log_2_FC values (2'-FL/Lac) obtained in the transcriptome analysis (a) are shown in parentheses. The genes of Fuc- and propanediol utilization clusters are enclosed with pink- and purple boxes, respectively. The genes in the propanediol utilization cluster can also be involved in a reductive glycerol utilization pathway. See the source data file for the individual data points corresponding to the graphs (b and c).

We then compared the transcriptomes of *C. perfringens* JCM 1290^T^ grown on 2'-FL and Lac. Among 2,982 genes, 267 were upregulated while 104 were downregulated by 2'-FL (|log_2_FC| > 1, P_adj_ < 0.01) (Figure (2a) and Supplementary Table S2). In addition to the Fuc utilization genes, several virulence-related genes for two extracellular GH33 sialidases (NanI and NanJ), α-toxin (PLC), and ethanolamine ammonia-lyase (EutABC), the latter two contributing to the progression of gas gangrene,^35,61^ were significantly upregulated. Alpha-toxin is also known as a potent activator of NLRP3 inflammasome.^27^ The increased transcription of the sialidase genes was accompanied by a significant increase in *N*-acetylneuraminic acid (NeuAc)-releasing activity from PGM (Figure (2b)), which indicates the relevance of transcriptome data. Several genes encoding GH families 29, 42, 85, and 112, which are presumably involved in host glycan breakdown,^29,32^ and the genes for the arginine deimination pathway, which counteracts pH drop by producing ammonia from arginine,^62^ were also induced. Consistent with the elevated expression of propanediol utilization genes, the strain produced a theoretically equimolar level of 1-propanol (19 mM) by metabolizing Fuc released from 2'-FL (1% ≈ 20 mM) (Figure (2c,d) and Supplementary Figure S7a). 1-Propanol was not detected when Lac was used as a carbon source. While a small amount of propionate (1.2 mM) was produced (Figure (1c) and Supplementary Figure S3b), neither 1,2- nor 1,3-propanediol was detected (Supplementary Figure S7b).^54^

**Figure 3.**
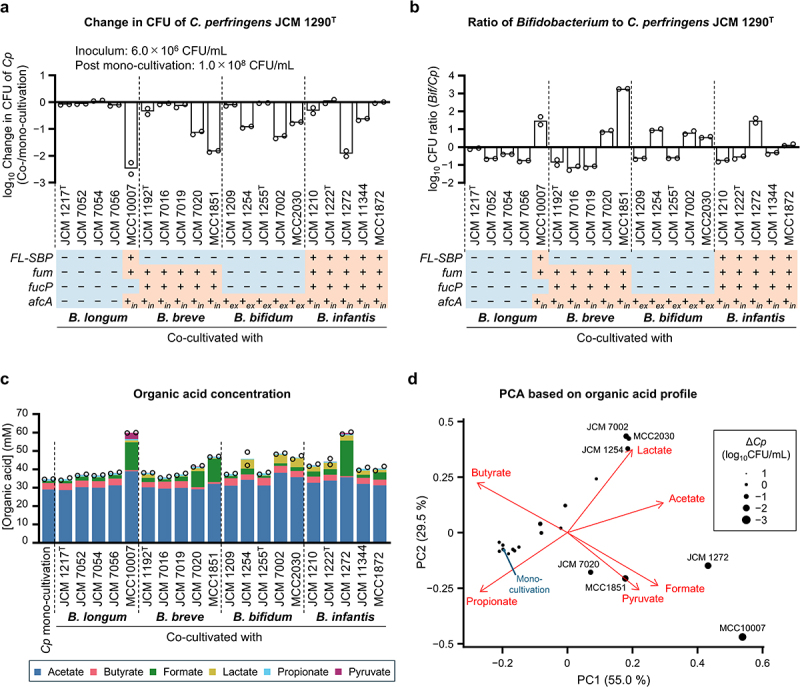
Varied ability of *Bifidobacterium* strains to reduce CFU of *C. perfringens* JCM 1290^T^ grown on 2'-FL. *C. perfringens* JCM 1290^T^ was co-cultivated with 20 different *Bifidobacterium* strains in YC medium supplemented with 0.5% 2'-FL for 24 h. a, change in CFU compared between post co-cultivation and post mono-cultivation of *C. perfringens* JCM 1290^T^. Log_10_ values are shown. The genotypes for FL-SBP (the solute-binding protein of FL transporter), Fum (cytoplasmic fuc metabolism), FucP (fuc permease), and AfcA (GH family 95 1,2-α-l-fucosidase) of *Bifidobacterium* strains are indicated. *in* and *ex* represent intracellular and extracellular localization of AfcA, respectively. b, ratio of post co-cultivation CFU of bifidobacteria to that of *C. perfringens* JCM 1290^T^. Log_10_ values are shown. Bars represent the mean of two independent experiments, with each data point shown by a circle (a and b). c, concentrations of acetate (blue), butyrate (pink), formate (green), lactate (khaki), propionate (cyan), and pyruvate (purple) in the supernatants post 24 h cultivation are shown in stacked bar charts. Neither valerate, iso-valerate, nor succinate was detected. Bars represent the mean of two independent experiments, with each data point indicating the total amount of detected organic acids (see supplementary figure 9 for the concentrations of individual organic acids). d, PCA plots based on the organic acid concentrations in the supernatants. Data obtained in (c) are used for plotting. Circle size represents the extent of decrease in CFU of *C. perfringens* after co-cultivation compared to mono-cultivation. Data shown in (a) were used for plotting. Arrows indicate the contribution of respective organic acids to the principal components. See the source data file for the individual data points corresponding to the graphs (a–c).

Given the potentially pathogenic growth of *C. perfringens* JCM 1290^T^ on 2'-FL, we examined the competitive ability of the genus *Bifidobacterium*, a dominant taxon in the infant gut,^7,11,63^ on this HMO. Strains belonging to the four prominent human gut-associated *Bifidobacterium* (sub)species, *i.e*., *B. longum*, *B. infantis*, *B. breve*, and *B. bifidum*, with varied 2'-FL assimilation phenotypes, were selected (Supplementary Figure S8). One *B. longum* strain (MCC10007) and five *B. infantis* strains, which possess FL transporter(s) and intracellular 1,2-α-l-fucosidases (GH family 95),^7,11^ showed considerable growth on 2'-FL, with their cell density ranging from OD_600_ 0.64 to 1.0. Three out of five strains of *B. bifidum*, which have an extracellular 1,2-α-l-fucosidase,^59^ also grew on 2'-FL. The marginal growth of JCM 1255^T^ on HMOs has been reported previously.^57^ JCM 1209 strain showed poor growth on all sugars tested. Four *B. longum* strains and five *B. breve* strains did not utilize 2'-FL due to the absence of FL transporters and extracellular 1,2-α-l-fucosidases. However, cell densities (OD_600_) of these strains at 24 h reached between 0.56 and 1.5, when grown in the medium supplemented with Fuc + Lac, by preferentially utilizing the latter (Supplementary Figure S8b). *B. breve* completely consumed Fuc in the presence of both Gal and Glc in the medium. Many strains of *B. longum* and *B. breve* lack Fuc transporter and Gal transporter genes, respectively.^11^ These twenty *Bifidobacterium* strains were used to challenge *C. perfringens* JCM 1290^T^ by inoculating a 2'-FL-supplemented YC medium with an initial CFU approximately one-tenth of that of *C. perfringens* (*Bifidobacterium* strains: 3.5–12 × 10^5^ CFU/mL; *C. perfringens*: 5.6–6.8 × 10^6^ CFU/mL). Post 24 h co-cultivation, growth (CFU) of the *C. perfringens* type strain was compared to that observed in mono-cultivation (Figure (3a)). *B. longum* MCC10007 was the most effective in reducing the CFU of *C. perfringens* JCM 1290^T^, followed by *B. infantis* JCM 1272 and *B. breve* MCC1851, with reductions ranging from 68- to 300-fold. The effect of *B. longum* MCC10007 and *B. infantis* JCM 1272 was expected as the strains can internalize 2'-FL directly. However, the effect of *B. breve* MCC1851 was surprising because the strain is unable to directly assimilate 2'-FL. As *C. perfringens* JCM 1290^T^ preferred Fuc over Lac while *B. breve* preferred Lac over Fuc when grown on Fuc plus Lac (Supplementary Figure S8b, c), Lac released by 1,2-α-l-fucosidase of *C. perfringens* JCM 1290^T^ could be cross-fed to *B. breve* MCC1851 (discussed later). The highest CFU ratio of *Bifidobacterium* to *C. perfringens* JCM 1290^T^ was obtained for *B. breve* MCC1851 (Figure (3b)). It is interesting to note that the decrease in CFU of *C. perfringens* JCM 1290^T^ was marginally detected for *B. breve* JCM 7016 and JCM 7019, which share the same genotype as MCC1851 regarding the metabolism of 2'-FL and the constituting sugars and exhibited comparable or higher growth on Fuc + Lac than MCC1851 (Figure (3a) and Supplementary Figure S8a). Intra-species variability was also observed for *B. infantis*. JCM 1210 and JCM 1222^T^ strains showed comparable growth on 2'-FL to JCM 1272, however, only JCM 1272 remarkably decreased the CFU of *C. perfringens* JCM 1290^T^. PCA of organic acid profiles observed in the culture supernatants at 24 h indicates that formate and pyruvate, which have low p*K*_a_ values, are associated with the bifidobacteria-mediated decrease in CFU of *C. perfringens* JCM 1290^T^, which resulted in the reduction of butyrate that bifidobacteria do not produce (Figure (3c, d) and Supplementary Figure S9). For the following experiments, we chose *B. longum* MCC10007 for its most effective ability to reduce CFU of *C. perfringens* JCM 1290^T^ and *B. breve* MCC1851 for its highest survival rate when co-cultivated with *C. perfringens* JCM 1290^T^.

In the time-series analysis, CFU per mL of *C. perfringens* JCM 1290^T^ remained above 2.5 × 10^7^ throughout the incubation periods during 24 h of mono-cultivation. In the co-cultivation experiments, CFU of the *C. perfringens* type strain increased to levels comparable to those observed in mono-cultivation at 6 h (*p* = 0.15, one-way ANOVA) (Supplementary Figure 10a, b); however, by 24 h, CFU significantly dropped to 0.05% of that obtained in mono-cultivation with statistical significance (1.0–1.6 × 10^4^ vs. 2.5 × 10^7^ CFU/mL). Estimated spore counts also significantly decreased by 31-fold and 1.8-fold post 24 h co-cultivation with *B. longum* MCC10007 and *B. breve* MCC1851, respectively, compared to those obtained in mono-cultivation (1.0 × 10^7^ and 1.8 × 10^8^ vs. 3.2 × 10^8^ CFU/mL). CFU of two *Bifidobacterium* strains increased up to 12 h. While CFU of *B. breve* MCC1851 remained high (5.4 × 10^8^ CFU/mL), that of *B. longum* MCC10007 dropped by 33,000-fold at 24 h compared to 12 h (5.2 × 10^8^ vs. 1.6 × 10^4^ CFU/mL). While most of the sugar was consumed when co-incubated with *B. longum* MCC10007, 33% of 2'-FL remained unconsumed when co-cultivated with *B. breve* MCC1851, which was in agreement with decreased organic acid concentrations in the supernatants and the attenuated pH drop (Supplementary Figure 10c – e).

**Figure 4.**
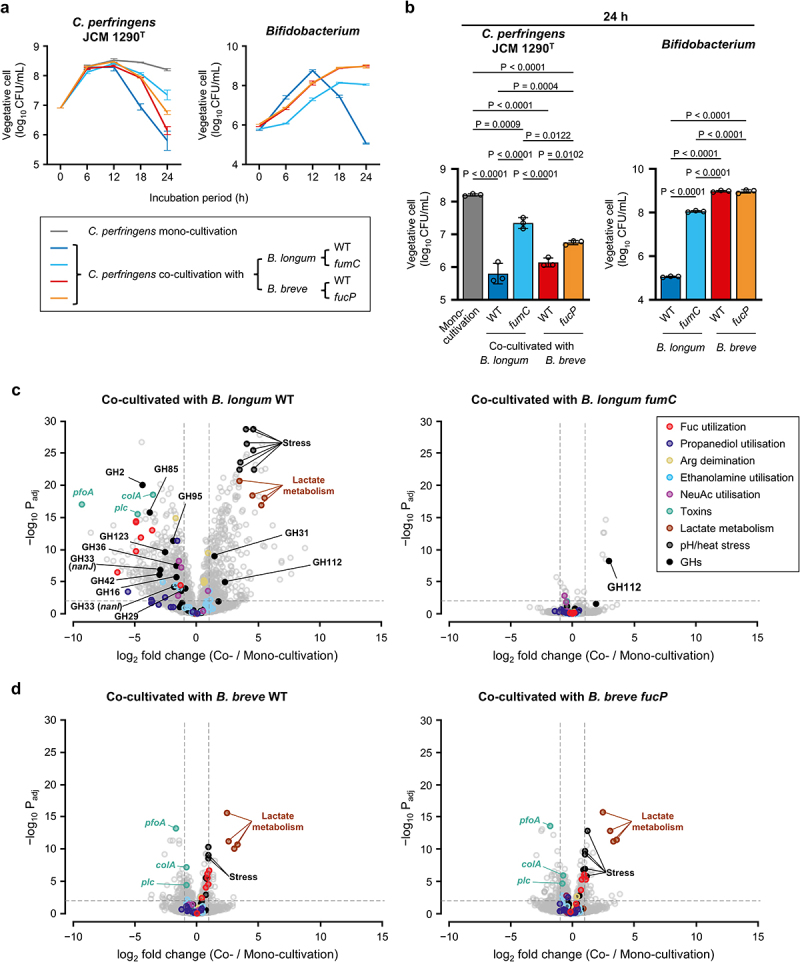
Selected *Bifidobacterium* strains impair a potentially pathogenic growth of *C. perfringens* JCM 1290^T^ on 2'-FL. a, time-dependent changes in CFU of *C. perfringens* JCM 1290^T^ during mono-cultivation (gray) and co-cultivation with *B. longum* MCC10007 or *B. breve* MCC1851 (left panel). WT (blue) and *fumC* (cyan) strains of *B. longum* MCC10007 and WT (red) and *fucP* (orange) strains of *B. breve* MCC1851 were used for co-cultivation. CFU of co-cultivated *Bifidobacterium* strains is also shown (right panel). 2'-FL was used as a carbon source. Data are mean ± SD of three biological replicates. b, CFU of *C. perfringens* JCM 1290^T^ and *Bifidobacterium* strains post 24 h co-cultivation is shown. Data in (a) were used. Bars and whiskers represent mean ± SD, with each data point shown by a circle. Tukey’s test following one-way ANOVA was used for statistical analysis. *p* values of less than 0.05 are indicated. c and d, volcano plots comparing the normalized gene expression values between the co-cultivated and mono-cultivated *C. perfringens* JCM 1290^T^ post 12 h cultivation. WT and *fumC* strains of *B. longum* MCC10007 (c) and WT and *fucP* strains of *B. breve* MCC1851 (d) were used for co-cultivation. Fold changes (log_2_) and adjusted *p* values (−log_10_) obtained by multiple *t*-tests with false discovery rate correction were used for plotting. Selected genes are shown in different colors with their functions or names. RNA-seq data of three biological replicates were used for the analysis (supplementary tables 1 and 7–10). See the source data file for the individual data points corresponding to the graphs (a and b).

**Figure 5.**
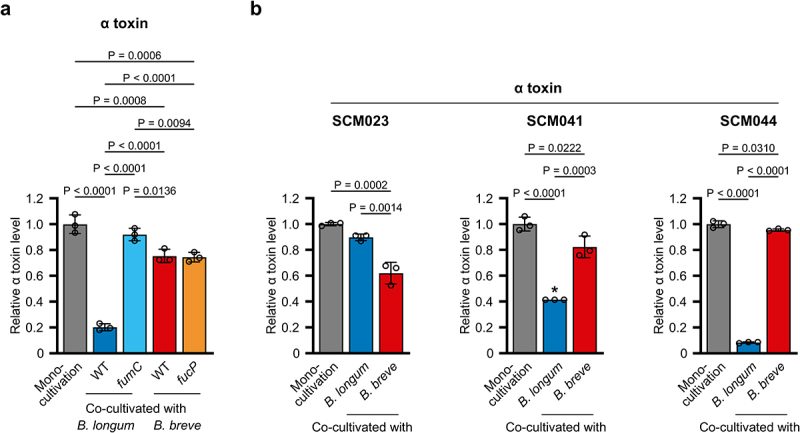
Alpha-toxin production by several *C. perfringens* strains upon co-cultivation with selected *Bifidobacterium* strains. a and b, relative α-toxin levels in the supernatants. Samples collected when cells were harvested for RNA-seq (figure 4c,d) were used for the assay (a). Three clinical *C. perfringens* strains were similarly co-cultivated with *B. longum* MCC10007 or *B. breve* MCC1851 (b). Bars and whiskers represent the mean ± SD of three independent experiments, with each data point shown by a circle. The asterisk indicates the lowest detection limit for α-toxin in the assay. Tukey’s test following one-way ANOVA was used for statistical analysis. *p* values of less than 0.05 are indicated. See the source data file for the individual data points corresponding to the graphs (a and b).

Transcriptome of *C. perfringens* JCM 1290^T^ was assessed using cells collected at 6- and 12 h co-cultivation on 2'-FL, in which wild-type (WT)- and *fumC* strains of *B. longum* MCC10007 or WT- and *fucP* strains of *B. breve* MCC1851 were used (Figure 4 and Supplementary Figure S11). We analyzed the transcriptome of cells harvested at these time points in anticipation of understanding how *C. perfringens* JCM 1290^T^ responds to the environmental changes induced by co-cultivation. The initial cell proliferation of the *C. perfringens* type strain during 6 h of co-incubation was reproduced when WT *Bifidobacterium* strains were used for co-cultivation. CFU of *C. perfringens* JCM 1290^T^ decreased by 24 h with statistical significance to similar extents that were observed in Supplementary Figure S10a. The estimated spore counts obtained post 24 h co-cultivation with *B. breve* MCC1851 did not show statistically significant difference compared to mono-cultivation. Profiles of sugar consumption and organic acid production were also quite similar between the two independent experiments (Supplementary Figures S10c – e vs. 11b – d). PCA demonstrated that transcription profiles of *C. perfringens* JCM 1290^T^ cluster together across samples collected at 6 h (Supplementary Figure 11e and Supplementary Tables S3–6). Remarkably, however, 336 genes and 529 genes of *C. perfringens* JCM 1290^T^ were up- and downregulated, respectively, upon co-cultivation with WT *B. longum* MCC10007 at 12 h, compared to mono-cultivation (|log_2_FC| > 1, P_adj_ <0.01) (Figure (4c) and Supplementary Table S7). Most of the virulence-related genes induced by 2'-FL in mono-cultivation were significantly downregulated by co-cultivation, which include the genes for host glycan-acting GHs, ethanolamine utilisation, and α-toxin (PLC). The α-toxin level in the co-culture supernatant significantly decreased to 20% compared to the mono-culture supernatant at 12 h (Figure (5a)). The decreased α-toxin production by *C. perfringens* JCM 1290^T^ cells upon co-cultivation with WT *B. longum* MCC10007 is also evident when the toxin level was normalized with the CFU of *C. perfringens* JCM 1290^T^ (Supplementary Figure S11f), indicating the relevance of the transcriptome data. In addition, *pfoA* and collagenase (*colA*) genes, whose expression was unchanged between the growth on 2'-FL and Lac (Figure (2a)), were significantly downregulated by 610- and 11-fold, respectively, in the type strain of *C. perfringens* (Figure (4c)). As mentioned above, PFO is another activator of NLRP3 inflammasome^27^ and is reported to be highly prevalent in the genomes of clinical *C. perfringens* strains isolated from infants diagnosed with NEC.^25^ On the other hand, the genes involved in the lactate metabolism and the pH- and heat-stress responses were significantly upregulated in *C. perfringens* JCM 1290^T^. Down-regulation of the *pfoA* gene and up-regulation of lactate metabolic genes were also observed when co-cultivated with the WT strain of *B. breve* MCC1851 for 12 h (Figure (4d) and Supplementary Table S8). Ferredoxin-dependent lactate oxidation is shown to cause ATP depletion in *Clostridioides difficile* cells by inhibiting ion gradient formation by the Rnf complex.^64^ As the genome of *C. perfringens* JCM 1290^T^ also contains the Rnf complex genes, upregulation of the lactate oxidation pathway could cause energy starvation in *C. perfringens* JCM 1290^T^ as well.

The *fumC* (Fuc dehydrogenase) gene disruption delayed the growth of *B. longum* MCC10007 on 2'-FL (Supplementary Figure S1d), which resulted in a 35-fold reduction in the ability to decrease CFU of *C. perfringens* JCM 1290^T^ compared to WT *B. longum* (Figure (4a, b)), although the effect remained statistically significant. Estimated spore counts (3.0 × 10^8^ CFU/mL) were comparable to those obtained in mono-cultivation (2.0 × 10^8^ CFU/mL) (Supplementary Figure S11a). The α-toxin was also detected at a level indistinguishable from that obtained in the mono-cultivation (Figure (5a)). Organic acid concentrations in the supernatant also decreased compared to co-cultivation with the WT strain, except for butyrate (Supplementary Figure S11c). The transcriptome profile of *C. perfringens* JCM 1290^T^ co-cultivated with the *fumC* mutant of *B. longum* MCC10007 was almost identical to that obtained post 12 h of mono-cultivation of the *C. perfringens* strain (Supplementary Figure S11e and Supplementary Table S9). When we incubated a revertant of the *fumC* mutant, in which a genomic rearrangement likely occurred at the locus by eliminating the integrated suicide plasmid (Supplementary Figure S1b, f, g), with *C. perfringens* JCM 1290^T^ for 24 h, the revertant strain regained the ability to reduce CFU of the *C. perfringens* strain to a level statistically indistinguishable from that of the original WT strain (Supplementary Figure S12). The revertant also reduced the estimated spore CFU counts compared to those in mono-cultivation, although the effect was slightly, but significantly, less pronounced than that observed with the WT strain. The results indicate the importance of an uninterrupted flow of sugar metabolism inside the cells for the efficient growth of *B. longum* MCC10007 on 2'-FL and the resulting competitive advantage over other microbes. By contrast, the *fucP* (Fuc permease) gene disruption did not drastically affect the ability of *B. breve* MCC1851 to reduce CFU of *C. perfringens* JCM 1290^T^, although CFU of the *C. perfringens* type strain was elevated by 4.0-fold with statistical significance compared to that obtained when co-cultivated with the WT strain (5.6 × 10^6^ vs. 1.4 × 10^6^ CFU/mL) (Figure (4b)). Estimated spore CFU counts (2.8 × 10^8^ CFU/mL) were comparable to those obtained for mono-cultivation (2.0 × 10^8^ CFU/mL) and co-cultivation with the WT strain (2.2 × 10^8^ CFU/mL). No marked changes were observed for the organic acid and sugar concentrations in the supernatants and the transcriptomes between *B. breve* WT- and *fucP* co-cultivated *C. perfringens* JCM 1290^T^, except for the residual Fuc concentration (Supplementary Figure 11c – e and Supplementary Table 10). Thus, the CFU-reducing effect of *B. breve* MCC1851 and likely JCM 7020 strains on *C. perfringens* JCM 1290^T^ (Figure (3a)) is largely attributed to Lac assimilation by the strains, which is dependent on 1,2-α-l-fucosidase of *C. perfringens* JCM 1290^T^. Decreased α-toxin production by *C. perfringens* JCM 1290^T^ was confirmed at the protein level during co-cultivation with both the WT and *fucP* strains of *B. breve* MCC1851 to a similar extent (~25%) (Figure (5a)). However, the difference was not significant for the *fucP*-co-cultivated *C. perfringens* JCM 1290^T^ when the toxin level was normalized with its CFU (Supplementary Figure 11f). The observed inconsistency in magnitude between the transcriptional and protein levels, including the results obtained from co-cultivation with WT *B. longum* MCC10007, might be attributed to the reported instability of α-toxin.^65^ Nonetheless, as the attenuation of the *plc* transcription is more pronounced during the co-cultivation with *B. longum* MCC10007 compared to co-cultivation with *B. breve* MCC1851 (27-fold vs. 1.7-fold, Figure (4c, d)), the effect on the normalized α-toxin level might be more clearly detected in the former case (Supplementary Figure S11f). The α-toxin production by three clinical isolates (SCM023, SCM041, and SCM044) also significantly decreased when co-cultivated with the WT strains of two *Bifidobacterium* species, except for *C. perfringens* SCM023 co-cultivated with *B. longum* (Figure (5b)). It should be noted that the strain SCM023 did not show a lagged growth compared to other strains such as JCM 1290^T^, SCM041, and SCM044 in 2'-FL-supplemented YC medium (Supplementary Figure S3a), and did not exhibit the preference for Fuc over Lac as opposed to JCM 1290^T^ (Supplementary Figure S8c). Thus, the ability of bifidobacteria to reduce α-toxin production and CFU of *C. perfringens* could differ not only among *Bifidobacterium* species and strains but also against different *C. perfringens* strains, depending on their sugar assimilation ability and preference. The expression level of Afc3 (1,2-α-l-fucosidase) could not only affect the growth of *C. perfringens* itself but also influence the competitive ability of *B. breve* strains.

There are several caveats to consider: our study does not involve *in vivo* experiments, and no clinically relevant adverse effects have been reported so far for 2'-FL administration to healthy adults and infants.^12–14^ In addition, a recent report mentioned an enhanced Muc2 expression in the colon of mice fed with 2'-FL.^66^ Nonetheless, we should be aware that many *C. perfringens* strains are able to assimilate 2'-FL to different degrees and that, at least for the type strain, the 2'-FL assimilation was accompanied by the upregulation of several virulence-related genes. Our study thus underscores the need for future experiments utilizing animal models with microbiota dominated by various *C. perfringens* strains to enhance the safety assessment of 2'-FL for clinical interventions.

## Note added in proof

During the revision process, a paper describing the degradation of sialylated HMOs by *C. perfringens* was published.^67^

## Supplementary Material

Supplemental Material

## Data Availability

The dataset generated in this study has been deposited in the DDBJ Sequence Read Archive (DRA) under the BioProject PRJDB17816.

## Code availability

All analyses were performed with publicly available software as mentioned above. All codes used in the analyses are provided in the Supplementary Methods.

## Material availability statement

All biological materials, except for human samples, are publicly available or will be distributed upon reasonable request.
