# Supplementary material for: In vitro competition with Bifidobacterium strains impairs potentially pathogenic growth of Clostridium perfringens on 2′-fucosyllactose
Source: Gut Microbes. 2025 Mar 18;17(1):2478306. doi: 10.1080/19490976.2025.2478306 (PMC11956901; doi:10.1080/19490976.2025.2478306)
Supplement: Supplemental Material [file KGMI_A_2478306_SM9322.zip › Suppmenentary Methods.docx]

# Supplementary Methods: codes used in this study

# R packages and external functions

A set of R packages was used for this analysis. The [pacman](https://cran.r-project.org/web/packages/pacman/index.html) package was used to simplify downloading and loading the required packages. All graphics and data wrangling were handled using the [tidyverse suite of packages](https://www.tidyverse.org/).

# install/load the pacman package for rapid installation of packages that are not in the library
if (!require("pacman")) install.packages("pacman")
# use pacman to install/load all packages needed for the analysis
pacman::p_load('tidyverse','tximport', 'gt','edgeR', 'matrixStats',
 'cowplot', 'pheatmap', 'Cairo', 'RColorBrewer', 'viridis')
set.seed(1992)

An external R function was used to keep the code tidy.

source("code/profile.R") # calculates counts per million (CPM) for each gene

# plots the distribution of CPM values for each sample
# this function accepts count data from a DGElist object, calculates counts per million (CPM) for each gene,
# and plots the distribution of CPM values for each sample
# input:
# (1) count data from a DGElist object
# (2) vector with sample names
# (3) string that will be used as a subtitle
# example: profile(myDGEList, sampleLabels, "Unfiltered, non-normalized")

profile <- function(data, samples, subtitle){
# use the 'cpm' function from EdgeR to get CPM
log2.cpm <- cpm(data, log=TRUE)
log2.cpm.df <- as_tibble(log2.cpm, rownames = "geneID") # existing rownames are transferred to column geneID
colnames(log2.cpm.df) <- c("geneID", samples)
# pivot data
log2.cpm.df.pivot <- pivot_longer(log2.cpm.df, # dataframe to be pivoted
 cols = -1, # select all columns except the first one to be stored as a SINGLE variable
 names_to = "samples", # name of that new variable (column)
 values_to = "expression") # name of new variable (column) storing all the values (data)
# plot the distribution of CPM values
ggplot(log2.cpm.df.pivot) +
 aes(x=samples, y=expression, fill=samples) +
 geom_violin(trim = FALSE, show.legend = FALSE) +
 stat_summary(fun = "median",
 geom = "point",
 shape = 124,
 size = 2.5,
 color = "black",
 show.legend = FALSE) +
 labs(y="log2 expression", x = "sample",
 title="Log2 Counts per Million (CPM)",
 subtitle=subtitle) +
 coord_flip() +
 theme_bw()
}

# Analysis of RNA-seq data, related to Figures 1, 2 and Supplementary Fig. 8

## Processing of raw fastq files and read mapping

The code chunk below describes the processing of raw FASTQ files and mapping reads to the *Clostridium perfringens* ATCC 13124 transcriptome. The following software is required:

1. [fastp (v0.22.3)](https://github.com/OpenGene/fastp)
2. [Bowtie2 (v 2.4.5)](http://bowtie-bio.sourceforge.net/bowtie2/index.shtml)
3. [Kallisto (v0.48)](https://pachterlab.github.io/kallisto/)
4. [MultiQC (v1.13)](https://multiqc.info/)
5. [Parallel (v20220722)](https://www.gnu.org/software/parallel/)

Put fastq.gz files to data/rnaseq/fastq/.

The reference FASTA files used for building Bowtie2 and Kallisto indices were put to data/rnaseq/refs/.

**Note**: exact file names (without the .fastq.qz extension) should be entered into data/rnaseq/runids.txt.

Summary of the script:

1. Quality control of raw reads was carried out using fastp
2. Quality trimming and removal of Illumina sequencing adapters via fastp
3. Reads were aligned against rRNA and tRNA gene sequences extracted from the *Clostridium perfringens* ATCC 13124 genome (GenBank accession no. NC_008261.1) using Bowtie2 to check the efficiency of rRNA depletion
4. Reads were mapped to the *Clostridium perfringens* ATCC 13124 transcriptome using Kallisto
5. The quality of raw reads, as well as the results of Bowtie2/Kallisto mapping were summarized in data/rnaseq/multiqc_report.html generated via MultiQC

source ~/.bash_profile
echo $BASH_VERSION
#set -ex

####################
## SOFTWARE SETUP ##
####################
# required tools: fastp (v0.23.2), bowtie2 (v2.4.4), kallisto (v0.48)
# multiqc (v1.13), and parallel (v20220722)
# set the name of conda environments
environment_name="transcriptomics"

################
## USER INPUT ##
################
# fastq files should be in data/rnaseq/fastq/
# txt file containing names of fastq files (without the fastq.gz extension)
sample_names="data/rnaseq/runids.txt"
# fasta file containing sequences of rRNA and tRNA genes
rRNA_tRNA_fasta="data/rnaseq/refs/Cperfringens_rRNA_tRNA.fasta"
# fasta file containing the whole transcriptome
transcriptome_fasta="data/rnaseq/refs/Cperfringens_transcriptome.fasta"
# name for the bowtie2 index
bowtie2_index_name="data/rnaseq/refs/Cperfringens_rRNA_tRNA"
# name for the kallisto index
kallisto_index_name="data/rnaseq/refs/Cperfringens_transcriptome.index"

# create directories
echo "Creating directories"
mkdir -p data/rnaseq/qc # qc results for raw reads
mkdir -p data/rnaseq/fq_trim # trimmed reads
mkdir -p data/rnaseq/sam # sam files produced during bowtie2 alignment; will be deleted
mkdir -p data/rnaseq/kallisto # kallisto mapping results

# activate conda environment
eval "$(command conda 'shell.bash' 'hook' 2> /dev/null)" # initializes conda in sub-shell
conda activate ${environment_name}
conda info|egrep "conda version|active environment"

# run fastp on raw reads
echo "Running fastp on raw reads"
cat ${sample_names} | parallel -j 50% "fastp \
-i data/rnaseq/fastq/{}_R1_001.fastq.gz \
-I data/rnaseq/fastq/{}_R2_001.fastq.gz \
-o data/rnaseq/fq_trim/{}_R1_tr.fastq.gz \
-O data/rnaseq/fq_trim/{}_R2_tr.fastq.gz \
-w 1 \
-h data/rnaseq/qc/{}_fastp.html \
-j data/rnaseq/qc/{}_fastp.json"

# check mapping to rRNA and tRNA
echo "Filtering reads that map to rRNA and tRNA using Bowtie2"
# build bowtie2 index
bowtie2-build ${rRNA_tRNA_fasta} \
${bowtie2_index_name}
# align reads via bowtie2; save ones that did not align to a separate file
cat ${sample_names} | \
parallel "bowtie2 -x ${bowtie2_index_name} \
-1 data/rnaseq/fq_trim/{}_R1_tr.fastq.gz \
-2 data/rnaseq/fq_trim/{}_R2_tr.fastq.gz \
-S data/rnaseq/sam/{}.sam \
&> data/rnaseq/fq_trim/{}.log"

# pseudolalign reads to transcriptome_fasta
echo "Mapping reads to the transcriptome via Kallisto"
# build kallisto index
kallisto index -i ${kallisto_index_name} \
${transcriptome_fasta}
# map reads to indexed reference via kallisto
cat ${sample_names} | parallel "kallisto quant \
-i ${kallisto_index_name} \
-o data/rnaseq/kallisto/{} \
--rf-stranded \
data/rnaseq/fq_trim/{}_R1_tr.fastq.gz data/rnaseq/fq_trim/{}_R2_tr.fastq.gz \
&> data/rnaseq/kallisto/{}_2.log"

# run multiqc
echo "Running MultiQC"
export LC_ALL=en_US.utf-8
export LANG=en_US.utf-8
multiqc -d data/rnaseq -o data/rnaseq

# remove directories with intermediate files
echo "Removing directories"
rm -rf data/rnaseq/qc
rm -rf data/rnaseq/fq_trim
rm -rf data/rnaseq/sam

## Analysis via the Kallisto pipeline

### Importing count data into R

[TxImport](https://bioconductor.org/packages/release/bioc/html/tximport.html) was used to read Kallisto output into the R environment.

*Note*: before running the code, double-check that file names in the file_names column in data/rnaseq/studydesign.txt are identical to file names in data/rnaseq/runids.txt.

# read the study design file
targets <- read_tsv("data/rnaseq/studydesign.txt")
# set file paths to Kallisto output folders with quantification data
files <- file.path("data/rnaseq/kallisto", targets$file_name, "abundance.tsv")
# check that all output files are present
all(file.exists(files))

## [1] TRUE

# use 'tximport' to import Kallisto output into R
txi_kallisto <- tximport(files,
 type = "kallisto",
 txOut = TRUE, # import at transcript level
 countsFromAbundance = "lengthScaledTPM")

# capture variables of interest from the study design
condition <- as.factor(targets$condition)
culture <- as.factor(targets$culture)
coculture <- as.factor(targets$coculture)
sugar <- as.factor(targets$sugar)
time <- as.factor(targets$time)
batch <- as.factor(targets$batch)
# capture sample labels for later use
sampleLabels <- targets$sample

# create a table with raw counts for GEO submission
raw_counts <- as.tibble(txi_kallisto$counts, rownames = "locus_tag")
colnames(raw_counts) <- c("geneID", sampleLabels)
write_tsv(raw_counts, "results/rnaseq/tables/raw_count_matrix.txt")

# use the gt package to produce the study design table
gt(targets) %>%
 cols_align(
 align = "left",
 columns = everything()
 )

| sample | file_name | condition | culture | coculture | sugar | time | batch |
| --- | --- | --- | --- | --- | --- | --- | --- |
| G1_12h_rep1 | alone_12h_A_S25 | alone_12h | mono | mono_culture | FL_1per | 12h | 1 |
| G1_12h_rep2 | alone_12h_B_S26 | alone_12h | mono | mono_culture | FL_1per | 12h | 1 |
| G1_12h_rep3 | alone_12h_C_S27 | alone_12h | mono | mono_culture | FL_1per | 12h | 1 |
| G1_6h_rep1 | alone_6h_A_S10 | alone_6h | mono | mono_culture | FL_1per | 6h | 1 |
| G1_6h_rep2 | alone_6h_B_S11 | alone_6h | mono | mono_culture | FL_1per | 6h | 1 |
| G1_6h_rep3 | alone_6h_C_S12 | alone_6h | mono | mono_culture | FL_1per | 6h | 1 |
| G2_12h_rep1 | 10007_12h_A_S28 | lon10007_12h | di | longum_WT | FL_1per | 12h | 1 |
| G2_12h_rep2 | 10007_12h_B_S29 | lon10007_12h | di | longum_WT | FL_1per | 12h | 2 |
| G2_12h_rep3 | 10007_12h_C_S30 | lon10007_12h | di | longum_WT | FL_1per | 12h | 2 |
| G2_6h_rep1 | 10007_6h_A_S13 | lon10007_6h | di | longum_WT | FL_1per | 6h | 1 |
| G2_6h_rep2 | 10007_6h_B_S14 | lon10007_6h | di | longum_WT | FL_1per | 6h | 1 |
| G2_6h_rep3 | 10007_6h_C_S16 | lon10007_6h | di | longum_WT | FL_1per | 6h | 1 |
| G3_12h_rep1 | fumC_mutant_12h_A_S31 | lon_fumC_12h | di | longum_mutant | FL_1per | 12h | 1 |
| G3_12h_rep2 | fumC_mutant_12h_B_S20 | lon_fumC_12h | di | longum_mutant | FL_1per | 12h | 1 |
| G3_12h_rep3 | fumC_mutant_12h_C_S21 | lon_fumC_12h | di | longum_mutant | FL_1per | 12h | 1 |
| G3_6h_rep1 | fumC_mutant_6h_A_S15 | lon_fumC_6h | di | longum_mutant | FL_1per | 6h | 1 |
| G3_6h_rep2 | fumC_mutant_6h_B_S17 | lon_fumC_6h | di | longum_mutant | FL_1per | 6h | 1 |
| G3_6h_rep3 | fumC_mutant_6h_C_S18 | lon_fumC_6h | di | longum_mutant | FL_1per | 6h | 2 |
| G4_12h_rep1 | 1851_12h_A_S34 | bre1851_12h | di | breve_WT | FL_1per | 12h | 1 |
| G4_12h_rep2 | 1851_12h_B_S35 | bre1851_12h | di | breve_WT | FL_1per | 12h | 1 |
| G4_12h_rep3 | 1851_12h_C_S36 | bre1851_12h | di | breve_WT | FL_1per | 12h | 1 |
| G4_6h_rep1 | 1851_6h_A_S19 | bre1851_6h | di | breve_WT | FL_1per | 6h | 2 |
| G4_6h_rep2 | 1851_6h_B_S32 | bre1851_6h | di | breve_WT | FL_1per | 6h | 1 |
| G4_6h_rep3 | 1851_6h_C_S33 | bre1851_6h | di | breve_WT | FL_1per | 6h | 1 |
| G5_12h_rep1 | fucP_mutant_12h_A_S37 | bre_fucP_12h | di | breve_mutant | FL_1per | 12h | 1 |
| G5_12h_rep2 | fucP_mutant_12h_B_S38 | bre_fucP_12h | di | breve_mutant | FL_1per | 12h | 1 |
| G5_12h_rep3 | fucP_mutant_12h_C_S39 | bre_fucP_12h | di | breve_mutant | FL_1per | 12h | 1 |
| G5_6h_rep1 | fucP_mutant_6h_A_S22 | bre_fucP_6h | di | breve_mutant | FL_1per | 6h | 1 |
| G5_6h_rep2 | fucP_mutant_6h_B_S23 | bre_fucP_6h | di | breve_mutant | FL_1per | 6h | 1 |
| G5_6h_rep3 | fucP_mutant_6h_C_S24 | bre_fucP_6h | di | breve_mutant | FL_1per | 6h | 1 |
| mG1_rep1 | Lac_A_S40 | Lac | mono | mono | Lac | NA | 1 |
| mG1_rep2 | Lac_B_S41 | Lac | mono | mono | Lac | NA | 1 |
| mG1_rep3 | Lac_C_S42 | Lac | mono | mono | Lac | NA | 1 |
| mG2_rep1 | FL_A_S43 | FL | mono | mono | FL_0.5per | NA | 1 |
| mG2_rep2 | FL_B_S44 | FL | mono | mono | FL_0.5per | NA | 1 |
| mG2_rep3 | FL_C_S45 | FL | mono | mono | FL_0.5per | NA | 1 |

### Filtering and normalization

myDGEList <- DGEList(txi_kallisto$counts)
# plot unfiltered, non-normalized CPM
p1 <- profile(myDGEList, sampleLabels, "Unfiltered, non-normalized")
# filter counts
cpm <- cpm(myDGEList)
keepers <- rowSums(cpm>1)>=3 # only keep genes that have cpm>1 (== not zeroes)
# in more than 3 samples (minimal group size)
myDGEList.filtered <- myDGEList[keepers,]
# plot filtered, non-normalized CPM
p2 <- profile(myDGEList.filtered, sampleLabels, "Filtered, non-normalized")
# normalize counts via the TMM method implemented in edgeR
myDGEList.filtered.norm <- calcNormFactors(myDGEList.filtered, method = "TMM")
# plot filtered, normalized CPM
p3 <- profile(myDGEList.filtered.norm, sampleLabels, "Filtered, TMM normalized")
# compare distributions of the CPM values
plot_grid(p1, p2, p3, labels = c('A', 'B', 'C'), label_size = 12)


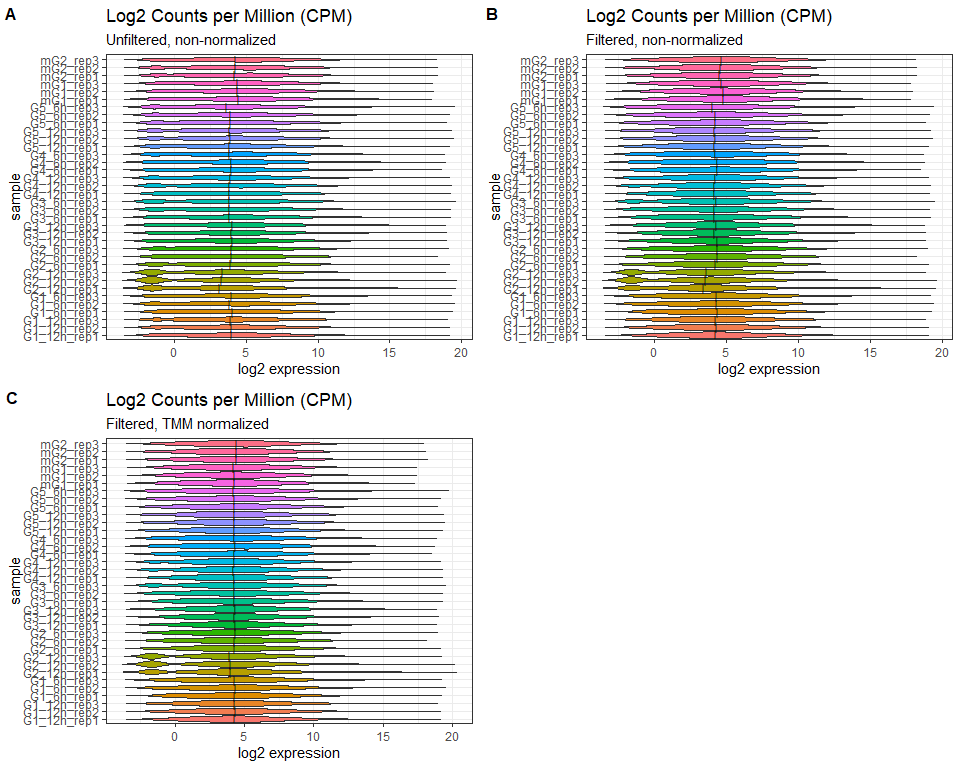


Filtering was carried out to remove lowly expressed genes. Genes with less than 1 count per million (CPM) in at least 3 or more samples were filtered out. This procedure reduced the number of genes from **2978** to **2743**. In addition, the [TMM method](https://doi.org/10.1186/gb-2010-11-3-r25) was used for between-sample normalization.

### PCA plot

Principal Component Analysis (PCA) plots reduce complex datasets to a 2D representation where each axis represents a source of variance (known or unknown) in the dataset.

# running PCA
log2.cpm.filtered.norm <- cpm(myDGEList.filtered.norm, log=TRUE)
pca.res <- prcomp(t(log2.cpm.filtered.norm[,1:30]), scale.=F, retx=T)
pc.var <- pca.res$sdev^2 # sdev^2 captures eigenvalues from the PCA result
pc.per <- round(pc.var/sum(pc.var)*100, 1) # calculate percentage of the total variation
# explained by each eigenvalue
# converting PCA result into a tibble for plotting
pca.res.df <- as_tibble(pca.res$x)

meta<-targets
meta$condition <- as.factor(targets$condition)
meta$culture <- as.factor(targets$culture)
meta$coculture <- as.factor(targets$coculture)
meta$sugar <- as.factor(targets$sugar)
meta$time <- as.factor(targets$time)
meta$batch <- as.factor(targets$batch)

meta$coculture<-factor(coculture,levels=c("mono_culture","longum_WT","longum_mutant","breve_WT","breve_mutant"))
meta$time<-factor(time,levels=c("6h","12h"))
library(ggplot2)
library(ggfortify)
autoplot(pca.res,data=meta[1:30,],shape="time",fill="coculture",color="coculture",size=6)+
 theme_classic()+
 scale_fill_manual(values=alpha(c("#bbbbbb","#0077bb","#33bbee","#DC050C","#F1932D"),0.5)) +
 scale_color_manual(values=c("#bbbbbb","#0077bb","#33bbee","#DC050C","#F1932D"))+
 scale_shape_manual(values=c(21,24))+
 guides(fill = guide_legend(override.aes=list(shape=21))) +
 xlab(paste0("PC1 (",pc.per[1],"%",")")) +
 ylab(paste0("PC2 (",pc.per[2],"%",")"))


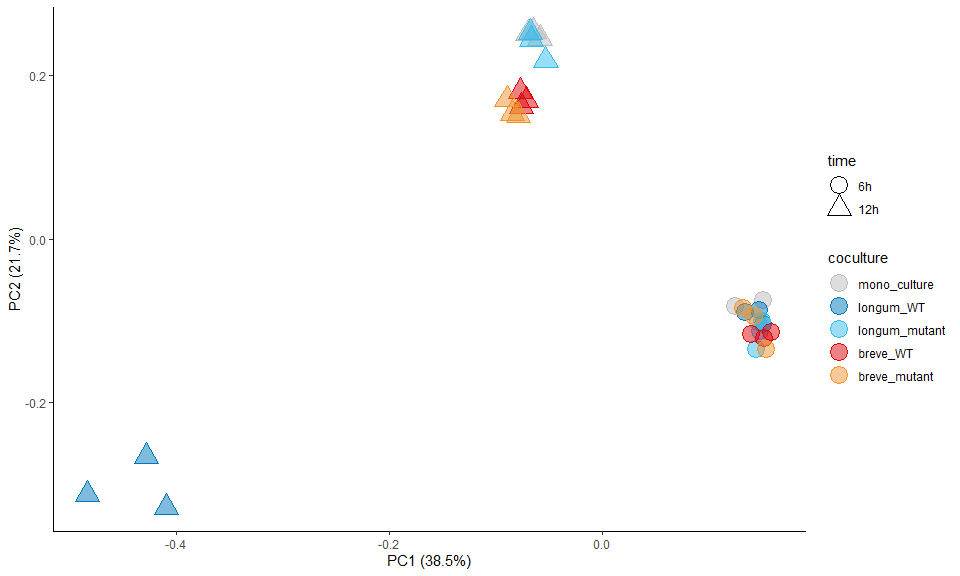


### Differentially expressed genes

To identify differentially expressed genes (DEGs), precision weights were first applied to each gene based on its mean-variance relationship using [VOOM](https://genomebiology.biomedcentral.com/articles/10.1186/gb-2014-15-2-r29). Linear modeling and bayesian stats were employed using [Limma](https://academic.oup.com/nar/article/43/7/e47/2414268) to find genes that were up- or down-regulated more than 2-fold at false-discovery rate (FDR) of 0.01.

# setting up model matrix without intercept
design <- model.matrix(~0 + condition)
colnames(design) <- levels(condition)
# using VOOM function from Limma package to apply precision weights to each gene
v.DEGList.filtered.norm <- voom(myDGEList.filtered.norm, design, plot = TRUE)


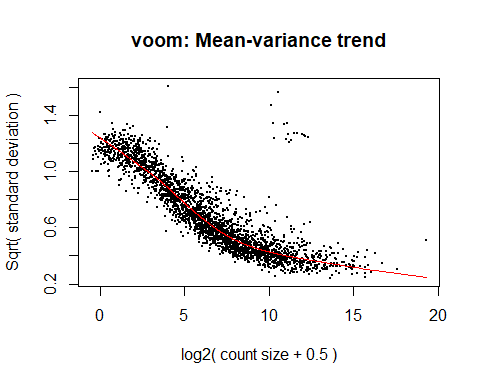


fit <- lmFit(v.DEGList.filtered.norm, design)
# setting up contrast matrix for pairwise comparisons of interest
contrast.matrix <- makeContrasts(FL_vs_Lac = FL - Lac,
 levels=design)
fits <- contrasts.fit(fit, contrast.matrix)
# extracting stats
ebFit <- eBayes(fits)

DEGs were annotated based on a [RAST-annotated](https://doi.org/10.1093/nar/gkt1226) version of the *Clostridium perfringens* ATCC 13124 genome, which was additionally subjected to extensive manual curation in mcSEED, a private clone of the publicly available [SEED platform](https://doi.org/10.1093/nar/gki866). The manual curation focused on annotating genes encoding functional roles (transporters, glycoside hydrolases, downstream catabolic enzymes, transcriptional regulators) involved in carbohydrate metabolism.

DEGs: *Clostridium perfringens* ATCC 13124 monoculture 2’FL vs. Lac

# create a master annotation table
seed.ann <- read_tsv('data/rnaseq/annotation/mcSEED_annotations.txt')
corr <- read_tsv('data/rnaseq/annotation/locus_tag_comparison.txt')
final.ann <- right_join(seed.ann, corr, by = c('seed_id' = 'seed_id')) %>%
 dplyr::select(locus_tag, annotation)

# annotate DEGs
myTopHits.FLvsLac <- topTable(ebFit, adjust ="BH", coef=1, number=3500, sort.by="logFC")
write.csv(myTopHits.FLvsLac,"myTopHits.FLvsLac.csv")

# setting up contrast matrix for pairwise comparisons of interest
contrast.matrix <- makeContrasts(breve_WT_6h_vs_alone_6h = bre1851_6h - alone_6h,
 breve_mut_6h_vs_alone_6h = bre_fucP_6h - alone_6h,
 longum_WT_6h_vs_alone_6h = lon10007_6h - alone_6h,
 longum_mut_6h_vs_alone_6h = lon_fumC_6h - alone_6h,
 levels=design)
fits <- contrasts.fit(fit, contrast.matrix)
# extracting stats
ebFit <- eBayes(fits)

DEGs: *Clostridium perfringens* ATCC 13124 co-culture *B. breve* 6h vs. monoculture 6h

# annotate DEGs
myTopHits.breve6hvs6h <- topTable(ebFit, adjust ="BH", coef=1, number=3500, sort.by="logFC")
write.csv(myTopHits.breve6hvs6h,"myTopHits.breve6hvs6h.csv")

DEGs: *Clostridium perfringens* ATCC 13124 co-culture *B. breve* mutant 6h vs. monoculture 6h

# annotate DEGs
myTopHits.breveMut6hvs6h <- topTable(ebFit, adjust ="BH", coef=2, number=3500, sort.by="logFC")
write.csv(myTopHits.breveMut6hvs6h,"myTopHits.breveMut6hvs6h.csv")

DEGs: *Clostridium perfringens* ATCC 13124 co-culture *B. longum* 6h vs. monoculture 6h

# annotate DEGs
myTopHits.longum6hvs6h <- topTable(ebFit, adjust ="BH", coef=3, number=3500, sort.by="logFC")
write.csv(myTopHits.longum6hvs6h,"myTopHits.longum6hvs6h.csv")

DEGs: *Clostridium perfringens* ATCC 13124 co-culture *B. longum* mutant 6h vs. monoculture 6h

# annotate DEGs
myTopHits.longumMut6hvs6h <- topTable(ebFit, adjust ="BH", coef=4, number=3500, sort.by="logFC")
write.csv(myTopHits.longumMut6hvs6h,"myTopHits.longumMut6hvs6h.csv")

# setting up contrast matrix for pairwise comparisons of interest
contrast.matrix <- makeContrasts(breve_WT_12h_vs_alone_12h = bre1851_12h - alone_12h,
 breve_mut_12h_vs_alone_12h = bre_fucP_12h - alone_12h,
 longum_WT_12h_vs_alone_12h = lon10007_12h - alone_12h,
 longum_mut_12h_vs_alone_12h = lon_fumC_12h - alone_12h,
 levels=design)

fits <- contrasts.fit(fit, contrast.matrix)
# extracting stats
ebFit <- eBayes(fits)

DEGs: *Clostridium perfringens* ATCC 13124 co-culture *B. breve* 12h vs. monoculture 12h

# annotate DEGs
myTopHits.breve12hvs12h <- topTable(ebFit, adjust ="BH", coef=1, number=3500, sort.by="logFC")
write.csv(myTopHits.breve12hvs12h,"myTopHits.breve12hvs12h.csv")

DEGs: *Clostridium perfringens* ATCC 13124 co-culture *B. breve* mutant 12h vs. monoculture 12h

# annotate DEGs
myTopHits.breveMut12hvs12h <- topTable(ebFit, adjust ="BH", coef=2, number=3500, sort.by="logFC")
write.csv(myTopHits.breveMut12hvs12h,"myTopHits.breveMut12hvs12h.csv")

DEGs: *Clostridium perfringens* ATCC 13124 co-culture *B. longum* 12h vs. monoculture 12h

# annotate DEGs
myTopHits.longum12hvs12h <- topTable(ebFit, adjust ="BH", coef=3, number=3500, sort.by="logFC")
write.csv(myTopHits.longum12hvs12h,"myTopHits.longum12hvs12h.csv")

DEGs: *Clostridium perfringens* ATCC 13124 co-culture *B. longum* mutant 12h vs. monoculture 12h

# annotate DEGs
myTopHits.longumMut12hvs12h <- topTable(ebFit, adjust ="BH", coef=4, number=3500, sort.by="logFC")
write.csv(myTopHits.longumMut12hvs12h,"myTopHits.longumMut12hvs12h.csv")

# Session info

The output from running ‘sessionInfo’ is shown below and details all packages necessary to reproduce the results in this report.

sessionInfo()

## R version 4.2.1 (2022-06-23 ucrt)
## Platform: x86_64-w64-mingw32/x64 (64-bit)
## Running under: Windows 10 x64 (build 19045)
##
## Matrix products: default
##
## locale:
## [1] LC_COLLATE=Japanese_Japan.utf8 LC_CTYPE=Japanese_Japan.utf8
## [3] LC_MONETARY=Japanese_Japan.utf8 LC_NUMERIC=C
## [5] LC_TIME=Japanese_Japan.utf8
##
## attached base packages:
## [1] stats graphics grDevices utils datasets methods base
##
## other attached packages:
## [1] viridis_0.6.4 viridisLite_0.4.2 RColorBrewer_1.1-3 Cairo_1.6-1
## [5] pheatmap_1.0.12 cowplot_1.1.1 matrixStats_0.63.0 edgeR_3.40.1
## [9] limma_3.54.0 gt_0.9.0 tximport_1.26.1 lubridate_1.9.2
## [13] forcats_1.0.0 stringr_1.5.0 dplyr_1.1.2 purrr_1.0.2
## [17] readr_2.1.4 tidyr_1.3.0 tibble_3.2.1 ggplot2_3.5.0
## [21] tidyverse_2.0.0 pacman_0.5.1 knitr_1.43 tinytex_0.46
## [25] rmarkdown_2.24
##
## loaded via a namespace (and not attached):
## [1] Rcpp_1.0.9 locfit_1.5-9.6 lattice_0.20-45
## [4] digest_0.6.30 utf8_1.2.2 R6_2.5.1
## [7] evaluate_0.21 highr_0.10 pillar_1.9.0
## [10] rlang_1.1.1 rstudioapi_0.15.0 labeling_0.4.2
## [13] bit_4.0.5 munsell_0.5.0 compiler_4.2.1
## [16] xfun_0.40 pkgconfig_2.0.3 htmltools_0.5.4
## [19] tidyselect_1.2.0 gridExtra_2.3 fansi_1.0.3
## [22] crayon_1.5.2 tzdb_0.4.0 withr_2.5.0
## [25] rhdf5filters_1.10.1 grid_4.2.1 gtable_0.3.4
## [28] lifecycle_1.0.3 magrittr_2.0.3 scales_1.3.0
## [31] cli_3.6.1 stringi_1.7.12 vroom_1.6.3
## [34] farver_2.1.1 xml2_1.3.5 generics_0.1.3
## [37] vctrs_0.6.3 Rhdf5lib_1.20.0 tools_4.2.1
## [40] bit64_4.0.5 glue_1.6.2 hms_1.1.3
## [43] parallel_4.2.1 fastmap_1.1.0 yaml_2.3.6
## [46] timechange_0.2.0 colorspace_2.0-3 rhdf5_2.42.1
