# Supplementary material for: In vitro competition with Bifidobacterium strains impairs potentially pathogenic growth of Clostridium perfringens on 2′-fucosyllactose
Source: Gut Microbes. 2025 Mar 18;17(1):2478306. doi: 10.1080/19490976.2025.2478306 (PMC11956901; doi:10.1080/19490976.2025.2478306)
Supplement: Supplemental Material [file KGMI_A_2478306_SM9322.zip › Supplementary_Figures.zip!/Supplementary Figures/Supplementary Figure Legends.docx]

**Supplementary Figure 1 | Construction of mutant *Bifidobacterium* strains and their phenotypic validation. a,** The metabolic pathway of 2′-FL utilization in *B. longum* MCC10007 (left) and *B. breve* MCC1851 (right) [11, 40, 41]. FL2, FL transporter-2; FucP, Fuc permerase; LacS, galactoside symporter; AfcA, 1,2-α-l-fucosidase; Bga2A, 1,4-β-galactosidase; FumC, Fuc dehydrogenase; FumD, l-fuconolactone hydrolase; FumE, l-fuconate dehydratase; FumF, 2-keto-3-deoxy-l-fuconate aldolase; FumG, lactaldehyde reductase. **b,** Schematic representation of the mutant construction via single crossover recombination. The primer annealing sites used for genomic PCR are indicated by arrow heads. The region used for homologous recombination is shown in light blue. **c,** Results of PCRs using WT and mutant genomic DNA as templates. The estimated amplicon sizes are 0.8- and 3 kbp for the WT and mutants, respectively. A molecular size marker (M) was loaded in the leftmost lane. **d,** Growth (OD_600_ values post 24 h cultivation) of the WT and mutant *Bifidobacterium* strains in YC medium without and with supplementation of sugars. Sugars added to the medium are: 0.5% Lac, 0.5% Fuc, 0.5% 2′-FL, 0.16% Fuc + 0.35% Lac, or 0.16% Fuc + 0.18% Gal + 0.18% Glc. Values obtained from biological duplicates are shown. The colour intensity indicates OD_600_ values. **e,** TLC analysis of sugars in the supernatants post 24 h cultivation on Fuc + Gal + Glc. *B. longum* MCC10007 lacks Fuc permease (*fucP*) and thus does not consume extracellular Fuc. *B. breve* MCC1851 WT strain consumes Fuc in the presence of both Glc and Gal (see **Supplementary Figure 8**). Representative results of biological duplicates are shown. **f** and **g,** Characterisation of revertants of the *fumC* mutant of *B. longum* MCC10007. See the Materials and Methods section for details on revertant isolation. The same primer pair as in (**c)** was used for the genomic PCR. A molecular size marker (M) was loaded in the leftmost lane (**f**). The sugar assimilation capability of the selected revertant was compared to that of the WT strain of *B. longum* MCC10007, by the method as in (**d**).

**Supplementary Figure 2 | Hydrolysis of 2′-FL by the recombinant CPF_RS10355 protein (1,2-α-l-fucosidase, Afc3) from *C. perfringens* JCM 1290^T^*.*** **a,** Domain structure of CPF_RS10355 is shown with the number of amino acid (aa) residues. The region corresponding to the catalytic domain (28–866 aa) was recombinantly expressed. The results of SDS-PAGE of the purified protein are shown on the right. **b,** An S-*v* plot of the hydrolysis of 2′-FL by the purified recombinant enzyme. Data of technical duplicates are shown, with the plots of a single technical replicate shown by green or purple circles. Kinetic parameters are the mean ± standard errors of duplicate experiments. See the source data file for the individual data points corresponding to the graph (**b**).

**Supplementary Figure 3 | Growth of *C. perfringens* strains on 2′-FL. a,** Growth of four JCM and three clinical *C. perfringens* strains in RCM (upper panel) or YC medium (lower panel) supplemented without (dashed green) and with 1% 2′-FL (pink). The growth was continuously monitored for 48 h by a plate reader in an anoxic chamber. Mean values (OD_605_) of biological duplicates are shown. The values obtained for the area under the curve (AUC) are shown in **Figure 1a**. **b,** Organic acid production by the seven *C. perfringens* strains grown in RCM or YC medium supplemented with 2′-FL. Concentrations of acetate, butyrate, formate, lactate, propionate, and pyruvate are shown. Neither valerate, iso-valerate, nor succinate was detected. Bars represent the mean of two independent experiments, with each data point shown by a circle (See **Figure 1c** for the total amount of the organic acids produced by each strain). See the source data file for the individual data points corresponding to the graphs (**a** and **b**).

**Supplementary Figure 4 | HMO degradation profile of *C. perfringens* strains**. **a,** Growth of four JCM and three clinical *C. perfringens* strains in YC medium supplemented without (dashed green) and with 1% HMOs purified from pooled milk (pink). Samples were taken for measuring OD_600_ values at the indicated time points. Line charts represent mean values of two biological replicates, with each data point shown by a circle. See the source data file for the individual data points corresponding to the graph. **b,** Degradation of each of HMOs during the growth. Data obtained from two independent experiments are shown. Colour intensity indicate the degradation of HMOs. Small amounts of Fuc, Gal, Glc, and Lac are present at 0 h as contaminants of the HMO mixture purified from pooled milk. Note that the elevated concentrations of HMOs (LNT and LN*n*T) at later incubation time compared to those obtained at 0 h or 12 h could result from the trimming of longer HMOs to the core structures or might be due to water evaporation from the medium during the incubation of small volume of the medium (SCM041 and SCM044 samples). 2′-FL: 2′-fucosyllactose; 3-FL: 3-fucosyllactose; DFL: difucosyllactose; LNT: lacto-*N*-tetraose; LN*n*T: lacto-*N*-*neo*tetraose; LNFP: lacto-*N*-fucopentaose; LNDFH: lacto-*N*-difucohexaose.

**Supplementary Figure 5 | Genotypying and toxinotyping of *C. perfringens* strains. a** and **b,** Four JCM and eight clinical strains were subjected to genomic PCR analysis. The genotypes for *afc3*, *pfoA*, and *eutB* (**a**) and the toxinotype for A–G (**b**) were determined. Results of agarose gel electrophoreses are shown, with DNA size markers loaded in the leftmost lanes. The primer annealing sites and the amplicon sizes for the respective genes are shown.

**Supplementary Figure 6 | The ability of five additionally isolated clinical *C. perfringens* strains to assimilate 2′-FL.** Five clinical *C. perfringens* strains, isolated from the faeces of five different infants, were additionally tested for their ability to utilise 2′-FL. **a** and **b,** Growth of the strains in RCM (upper panel) or YC medium (lower panel) supplemented without (dashed green) and with 1% 2′-FL (pink). The growth was continuously monitored by a plate reader in an anoxic chamber. The mean OD_605_ values obtained from two independent experiments are shown in (**a**). The areas under the curves (AUC, arbitrary unit) are shown in (**b**), with each data point shown by a circle. **c,** TLC analysis of sugars in supernatants of the *C. perfringen*s strains grown on 2′-FL for 48 h. Samples obtained in (**a**) were used for the analysis. Representative results of two biological replicates are shown. See the source data file for the individual data points corresponding to the graphs (**a** and **b**).

**Supplementary Figure 7 | *C. perfringens* JCM 1290^T^ produces 1-propanol by metabolising Fuc during the growth on 2′-FL. a** and **b,** Enlarged HPLC chromatograms detecting 1-propanol (**a**) and 1,2- and 1,3-propanediol (**b**). Post 24 h-culture supernatant of *C. perfringens* JCM 1290^T^ grown on 1% 2′-FL (≈ 20 mM) or Lac was used for the analysis. Standard alcohols were also co-injected with the supernatants for verification. Arrowheads indicate the standard compounds. Representative results of three biological replicates are shown. See the source data file for the individual data points corresponding to the graphs (**a** and **b**).

**Supplementary Figure 8 | Sugar fermentation capabilities of *Bifidobacterium* and *C. perfringens* strains. a,** Growth of *Bifidobacterium* strains in YC medium supplemented with 2′-FL or 2′-FL-constituting sugars. Saccharides added to the medium are: 0.5% Lac, 0.5% Fuc, 0.5% 2′-FL, 0.16% Fuc + 0.35% Lac, or 0.16% Fuc + 0.18% Gal + 0.18% Glc. OD_600_ values obtained post 24 h cultivation in two independent experiments are shown. The colour intensity indicates OD_600_ values. **b** and **c,** TLC analysis of sugars in the culture supernatants of *Bifidobacterium* strains (**b**) and *C. perfringens* strains (**c**) post 24 h cultivation on Fuc + Lac (upper panel) and Fuc + Gal + Glc (lower panel). The results shown are representative of two independent experiments. Note that all of the *B. breve* strains preferentially utilised Lac when grown on Fuc + Lac, while they utilised Fuc and Glc and left Gal when grown on Fuc + Gal + Glc (**b**). Preference for sugars also varies among the tested *C. perfringens* strains. JCM 1290^T^, AN685, and AN689 strains preferentially consumed Fuc over Lac when grown on Fuc + Lac, while the other strains appeared to utilise both of them equally or left Fuc unconsumed to varying extents. JCM 1290^T^ and JCM 3817 strains consumed all of the monosaccharides when grown on Fuc + Gal + Glc, while the other strains consumed Gal to varying extents. JCM 3816, SCM023, SCM401, AN673, AN680, and AN685 left Fuc unconsumed to different degrees.

**Supplementary Figure 9 |** **Organic acids in the supernatants post 24 h co-cultivation of *C. perfringens* JCM 1290^T^ with *Bifidobacterium* strains on 2′-FL.** Concentrations of acetate, butyrate, formate, lactate, propionate, and pyruvate are shown (See **Figure 3C**). Organic acids produced by mono-cultivation of *C. perfringens* JCM 1290^T^ on 2′-FL were also quantified. Neither valerate, iso-valerate, nor succinate was detected. Bars represent the mean of two independent experiments, with each data point shown by a circle. See the source data file for the individual data points corresponding to the graph.

**Supplementary Figure 10 | Time-series analysis of co-cultivation of *C. perfringens* JCM 1290^T^ with selected *Bifidobacterium* strains on 2′-FL. a,** Change in CFU of *C. perfringens* JCM 1290^T^ during mono-cultivation (grey) and co-cultivation with *B. longum* MCC10007 (blue) or *B. breve* MCC1851 (red) in YC medium supplemented with 0.5% 2′-FL (left panel). CFU of co-cultivated *Bifidobacterium* strains was also determined (right panel). CFU of *C. perfringens* JCM 1290^T^ at 6 h was not significantly different among mono- and co-cultivations (one-way ANOVA). **b,** CFU at 24 h mono- and co-cultivation is shown. Bars and whiskers represent the mean ± SD of three independent experiments, with each data point shown by a circle. Data in (**a**) were used. Spore CFU counts of *C. perfringens* JCM 1290^T^ were determined as described in the Materials and Methods section. Tukey’s test following one-way ANOVA (for *C. perfringens* JCM 1290^T^) or two-tailed *t*-test (for *Bifidobacterium*) was used for statistical analysis. P values of less than 0.05 are indicated. **c,** Change in pH of the culture supernatants. **d** and **e,** Changes in the concentrations of organic acids (**d**) and saccharides (**e**) in the culture supernatants. Neither valerate, iso-valerate, nor succinate was detected in (**d**). Gal and Glc were not detected in (**e**). Mean ± SD of three independent experiments are shown (**a** and **c–e**). The same colours as in (**a**) are used for (**b–e**). See the source data file for the individual data points corresponding to the graphs (**a–e**).

**Supplementary Figure 11 | Time-series analysis of co-cultivation of *C. perfringens* JCM 1290^T^ with WT and mutant *Bifidobacterium* strains on 2′-FL. a,** Spore CFU counts of *C. perfringens* JCM 1290^T^ post 24 h mono-cultivation (grey) and co-cultivation with bifidobacteria. WT (blue) and *fumC* (cyan) strains of *B. longum* MCC10007 and WT (red) and *fucP* (orange) strains of *B. breve* MCC1851 were used for co-cultivation. 2′-FL (0.5%) was used as a carbon source. Bars and whiskers represent the mean ± SD of three independent experiments, with each data point shown by a circle. **b,** pH of the culture supernatants. **c** and **d,** Changes in the concentrations of organic acids (**c**) and saccharides (**d**) in the culture supernatants. Neither valerate, *iso*-valerate, nor succinate was detected in (**c**). Gal and Glc were not detected in (**d**). Mean ± SD of three independent experiments is shown (**b**–**d**). Note that the data for WT strains shown in (**a–d**) were obtained in independent experiments from those shown in **Supplementary Figure 10. e,** PCA plots based on transcriptomes of *C. perfringens* JCM 1290^T^ post 6- (circle) and 12 h (triangle) mono-cultivation and co-cultivations. Note that up to eight genes were differentially expressed within the transcriptomes obtained at 6 h (see **Supplementary Tables 3–6**). **f,** Alpha-toxin levels in the supernatants (see **Figure 5a**) were normalised with the average CFU of *C. perfringens*. Samples taken at post 12 h cultivation were used for the analysis. Bars and whiskers represent the mean ± SD of three independent experiments, with each data point shown by a circle. The same colours as in (**a**) are used for (**b−f**). Tukey’s test following one-way ANOVA was used for statistical analysis (**a** and **f**), and P values of less than 0.05 are indicated. See the source data file for the individual data points corresponding to the graphs (**a–d** and **f**).

**Supplementary Figure 12 | Co-cultivation of *C. perfringens* JCM 1290^T^ with WT and *fumC* revertant strains of *B. longum* MCC10007.** CFU of *C. perfringens* JCM 1290^T^ (left panel) and *B. longum* MCC10007 (center panel) post 24 h mono-cultivation (grey) and co-cultivation with WT (blue) and *fumC* revertant (green) strains of *B. longum* MCC10007 in YC medium supplemented with 0.5% 2′-FL is shown. Spore CFU counts of *C. perfringens* JCM 1290^T^ (right panel) are also shown. Spore CFU counts of *C. perfringens* JCM 1290^T^ were determined as described in the Materials and Methods section. Bars and whiskers represent the mean ± SD of three independent experiments, with each data point shown by a circle. Tukey’s test following one-way ANOVA (for *C. perfringens* JCM 1290^T^) or two-tailed *t*-test (for *B. longum*) was used for statistical analysis. P values of less than 0.05 are indicated. See the source data file for the individual data points corresponding to the graph.
